# Implication of LAMP proteins and autophagy markers in colorectal cancer aggressiveness

**DOI:** 10.3389/fimmu.2025.1662830

**Published:** 2025-09-30

**Authors:** Tsvetomira Ivanova, Dorian Dikov, Diana Molander, Angel M. Dzhambov, Yordan Sbirkov, Nikolay Mehterov, Nikolay Belev, Boyko Atanasov, Maria Kazakova, Victoria Sarafian

**Affiliations:** ^1^ Department of Medical Biology, Medical University-Plovdiv, Plovdiv, Bulgaria; ^2^ Research Institute at Medical University-Plovdiv, Plovdiv, Bulgaria; ^3^ Department of Pathology, Jossigny Hospital, Jossigny, France; ^4^ Environmental Health Division, Research Institute at Medical University of Plovdiv, Plovdiv, Bulgaria; ^5^ Department of Propaedeutics of Surgical Diseases, Medical University-Plovdiv, University Hospital Eurohospital, Plovdiv, Bulgaria; ^6^ Department of Propaedeutics of Surgical Diseases, Medical University-Plovdiv, Unihospital, Panagyurishte, Bulgaria

**Keywords:** LAMP1, LAMP2, BECLIN1, LC3B, tumor budding, liquid biopsy

## Abstract

Lysosome-associated membrane proteins (LAMPs) play a critical role in various cellular processes, including phagocytosis, lipid transport, neoangiogenesis, and tissue remodeling. Recent discussions have focused on their involvement in autophagy related to tumor progression. Emerging studies have underscored the connection between tumorigenesis and autophagy, highlighting that dysregulation of this process is associated with the resistance of tumor cells to conventional chemotherapy across numerous malignancies, including colorectal cancer (CRC). Currently, there is a notable absence of reliable prognostic biomarkers for effectively stratifying and predicting treatment responses in CRC patients. Our study examines the expression of LAMP1 and LAMP2 proteins and genes alongside key autophagy markers such as BECLIN1 and LC3B, investigating their relationships with CRC invasiveness. We present original data illustrating the association of LAMP molecules with standard autophagy markers and tumor budding. Our findings provide novel insights into the significance of these markers in CRC invasiveness, their relationship with survival rates, and their potential role as prognostic biomarkers.

## Graphical Abstract

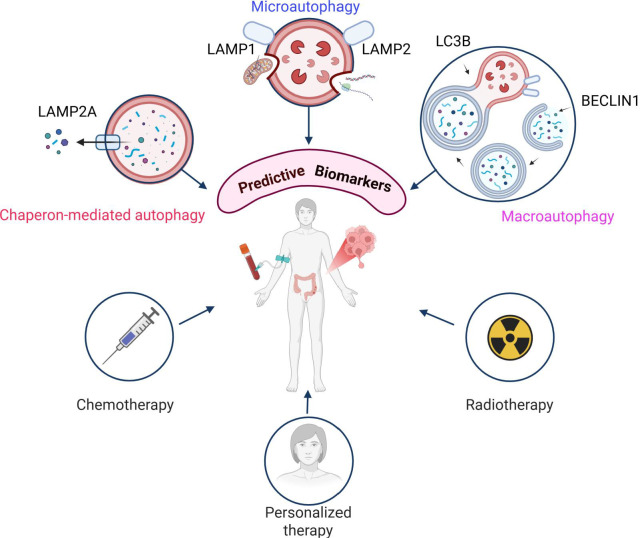

## Introduction

Colorectal cancer (CRC) is a major contributor to cancer related mortality and morbidity. It is the third most common type of cancer and the second leading cause of cancer-related deaths worldwide. By 2040, the number of CRC cases is predicted to reach 3.2 million (1). Despite the availability of therapeutic options, there remains a lack of reliable evidence to guide optimal treatment strategies. In the era of personalized medicine, high interpatient and intratumoral heterogeneity pose significant challenges to effective disease management.

Histopathological analysis remains essential for both clinical management and oncology research. Biopsy plays a crucial role in detecting and monitoring early relapses. In addition, molecular profiling provides essential information that guides subsequent decisions and predicts therapeutic response in CRC (2). One key histopathological feature is tumor budding, characterized by the dissociation of tumor cells from the primary tumor mass. observed in various types of cancers. It is defined as the presence of single tumor cells or small clusters of up to four cells located at the invasive tumor front (3). Increasing evidence suggests that tumor budding is associated with epithelial-mesenchymal transition (EMT), contributing to tumor aggressiveness. Although considered a poor prognostic factor for CRC (3), tumor budding has yet to be incorporated into routine pathology reporting.

Recent studies have increasingly highlighted the link between tumorigenesis and autophagy. Autophagy is a crucial catabolic process responsible for degrading redundant or damaged cellular components, thereby maintaining cellular homeostasis (4). It plays a fundamental role in energy metabolism, macromolecular synthesis, and the clearance of potentially harmful elements. In cancer, autophagy exerts a dual role—acting as both a tumor suppressor and a tumor promoter, depending on the stage of tumor progression (5). In early tumor development, autophagy facilitates removal of damaged organelles and toxic waste, promoting tumor cell death (6, 7). Conversely, in advanced stages, it serves as a survival mechanism enabling tumor cells to adapt to metabolic stress hypoxia, and nutrient deprivation within the tumor microenvironment (TME), as well as contributing to therapy resistance (8). Dysregulated autophagy has been implicated in both CRC carcinogenesis and resistance of tumor cells to conventional therapeutic approaches. Several studies have demonstrated that inhibition of autophagy can enhance the effectiveness of 5-FU chemotherapy in CRC in both *in vitro* and *in vivo* models (9). Moreover, autophagy activation during chemotherapy, has been shown to promote cancer cell death by blocking apoptosis (10).

The three main types of autophagy, microautophagy, macroautophagy, and chaperon–mediated autophagy (CMA) share a common target, through mechanistically different selective pathways. The lysosome plays a central role in all three processes. Among the five known lysosome-associated membrane proteins, LAMP1 and LAMP2 are the most widely expressed. These glycoproteins are implicated in a multitude of cellular processes, including phagocytosis, lipid transport, and aging. They are also believed to facilitate neoangiogenesis, tissue remodeling and tumor progression through interactions with extracellular matrix proteins (11). Despite the numerous studies, the precise functions remain incompletely understood. In recent years, increasing evidence has linked LAMP1 and LAMP2 to autophagy (12–16). Notably, studies have highlighted a significant role for the LAMP2A isoform, which is essential for CMA (17, 18). Additionally, LAMP2B has been shown to facilitate autophagosome-lysosome fusion in cardiomyocytes (19, 20), and LAMP2C is implicated in RNA and DNA autophagy by direct nucleic acid degradation (21–23).

Another key autophagy marker, BECLIN1 is frequently overexpressed in tumor cells and is often associated with poor prognosis (24). BECLIN1 is involved in the formation of the autophagosome (25) and makes the first connection between autophagy and tumor cells (26). Similarly, LC3 (microtubule-associated protein 1 light chain 3), a microtubule-associated protein, plays a crucial role in the progression of autophagy (27). The cleavage of the cytosolic form of LC3 yields three variants, among which membrane-associated LC3B is the most closely associated with autophagy (27). Dysregulated levels of LC3 have been implicated in various malignancies, including brain cancer, CRC, and melanoma (28, 29).

All these studies provide significant evidence supporting the potential use of LAMP proteins as novel predictive markers or therapeutic targets, given that autophagy plays a pivotal role in CRC development and response to therapy. Currently, there is a critical need for reliable prognostic biomarkers to improve patient stratification and treatment responses prediction in CRC patients. Therefore, our study aimed to investigate the expression of LAMP1 and LAMP2 at both the protein and gene levels, in parallel with the key autophagy markers BECLIN1 and LC3B, and their relationship with the invasiveness of CRC. By analyzing patient tissue and blood samples using immunohistochemistry, ELISA, and qPCR, we provide original data on the association between LAMP molecules, standard autophagy markers, and tumor budding. This novel data sheds light on the role these molecules play in the invasiveness of CRC. Additionally, they are linked to survival rates and hold potential as prognostic biomarkers.

## Materials and methods

### Patients and samples

Samples of clinically and histologically diagnosed CRC patients in the Centre Hospitalier de Marne-la Vallee, France (n=31) and in the Department of General and Clinical Pathology, Medical University of Plovdiv, Bulgaria (n=48), in the period of 2021-2024, were included in the study. Medical records provided patient demographic information (age, sex) and clinicopathological data, including tumor localization, histological grade, tumor stage - pTNM, lymphovascular invasion, presence of mutations, microsatellite instability (MSI) and tumor budding.

Tissue samples from CRC patients were examined in parallel with control samples. The internal control consisted of fifteen normal colonic tissues obtained from regions distal to the tumor site of CRC patients. External control included 5 non-neoplastic colon tissue samples (age-matched to CRC patients), derived from surgical margins of specimens from patients with sigmoid diverticulosis.

The resected colonic tissue was cryopreserved in a vial of liquid nitrogen, while another piece was fixed in 10% neutral buffered formaldehyde and embedded in paraffin. 4µm thick paraffin sections were cut for immunohistochemical analysis and hematoxylin-eosin (HE) staining. Surgical specimens were histologically evaluated by two independent pathologists, following the 8^th^ edition of the American Joint Committee on Cancer guidelines (30).

Peripheral blood was collected from the CRC patients prior to surgery, as well as from healthy controls. Plasma and white blood cells (WBCs) were isolated and stored at − 80 °C for subsequent analyses.

The study was approved by the Ethics Committee of the Medical University of Plovdiv (Protocol No 4/08.06. 2022), and informed consent was obtained from all participants. None of the patients had received prior radio therapy or neoadjuvant therapy.

### Immunohistochemistry

Immunohistochemistry was performed using the Novocastra™ Peroxidase Detection System (Cat No RE7110-K). After antigen retrieval, endogenous peroxidase was blocked followed by protein blocking. All samples were incubated with primary anti-human LAMP1 and LAMP2 mouse monoclonal antibodies (clones H4A3 and H4B4, DSHB, Yowa University) at working dilution of 1:100; anti-LAMP2A (Abcam Cat. No Ab125068) at a dilution of 1:100; anti- Beclinazako1 (GeneTex Cat. No GTX31722) at a dilution of 1:300; anti- LC3B (GeneTex Cat. No GTX82986) at a dilution of 1:400 at 4°C, overnight. All samples were then treated with secondary antibodies and DAB chromogen as previously described (31). The specificity of staining was assessed using negative controls as shown by Ivanova et al. (31). Immunoreactivity was assessed by using a semi-quantitative scale evaluating the expression in all cells in one visual field at 400 X magnification: 3+ (80-100% positive tumor cells), 2+ (40-80% positive tumor cells), 1+ (20-40% positive tumor cells), and (-) for no reaction.

### Tumor budding assessment

**Figure 1:**
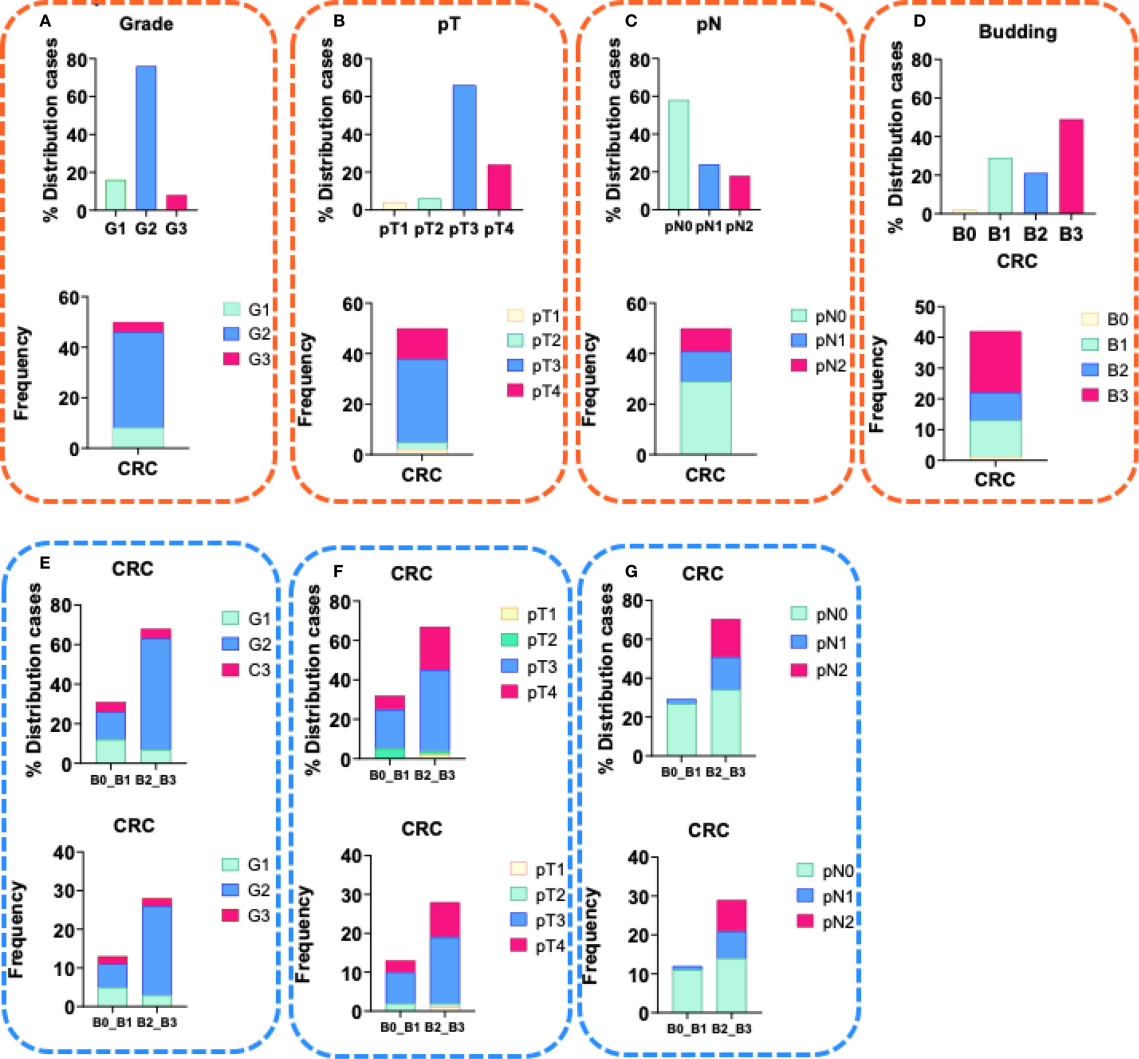
Characteristics of CRC patients. **(A)** Distribution (%) and frequency of CRC patients with tumor grade of differentiation G1- well-differentiated tumor, G2- moderately differentiated tumor and G3- poorly differentiated tumor, in the study group; **(B)** Distribution (%) and frequency of CRC patients with tumor size pT1, pT2, pT3 and pT4 within the study group; **(C)** Distribution (%) and frequency of CRC patients with invasion in the lymph nodes pN0, pN1and pN2 within the study group; **(D)** Distribution (%) and frequency of CRC patients with tumor budding B0, B1, B2 and B3 within the study group; **(E)** Distribution (%) and frequency of CRC patients with tumor grade of differentiation G1- well-differentiated tumor, G2- moderately differentiated tumor and G3- poorly differentiated tumor, in group with budding B0+B1 and in group with budding B2+B3; **(F)** Distribution (%) and frequency of CRC patients with tumor size pT1, pT2, pT3 and pT4 within the study group; **(G)** Distribution (%) and frequency of CRC patients with invasion in the lymph nodes pN0, pN1and pN2 within the study group.

A senior pathologist (DD) conducted a quantitative morphologic analysis of tumor budding, according to the guidelines outlined in the International Tumor Budding Consensus Conference (ITBCC) of 2016. The analysis involved scoring tumor budding using a 3-tier system (Bd1-Bd3), based on the number of buds observed in the highest count after examining 10 separate fields (at 20x objective lens) along the invasive front of the tumor. The assessment of tumor buds was based on HE evaluation (32) and the results were corrected for microscope eyepiece field diameter and bud count normalization to a field area of 0.785 mm2 (**Figure 1B**).

### ELISA

Plasma protein levels of LAMP1, LAMP2 and BECLIN1 of both CRC patients and controls were assessed by ELISA using commercial kits (DEVELOP Cat No. DL-LAMP1-Hu and Cat No. DL-LAMP2-Hu; Cloud-Clon Corp, Cat No. SEJ557Hu) according to the manufacturer’s protocols. All samples were analyzed in duplicate, and absorbance was measured at 405 nm using the ELISA Sunrise Reader (Tecan, Mannedorf, Switzerland).

### RNA extraction and gene expression analysis

Total RNA was extracted from WBCs using TRIzol (ThermoFisher Scientific, USA, Cat No. 1559602). DNA contamination was removed by TURBO DNA-free™ treatment (TURBO DNA-free kit, Thermo Fisher Scientific, USA, Cat No. AM1907). RNA concentration and purity were measured spectrophotometrically with NanoDrop 2000 (Thermo Scientific, USA). Gene expression in WBCs was assessed using the RevertAidTM First-strand cDNA Synthesis Kit (Thermo Fisher Scientific, USA, Cat. No. K1632) to obtain cDNA.

qRT-PCR analysis was performed using the Green MasterMix, (Genaxxon bioscience GmbH, Ulm, Germany, Cat No. M3023.0500) according to the manufacturer’s instructions. Each sample was run in duplicate for analysis on a Rotor-Gene Q 600 real-time PCR detection system (Qiagen, Hilden, Germany). The data were normalized to GAPDH, ACTINB, and UBC as endogenous controls. The primer sequences for all genes are listed in **Table 1A**. Relative gene expression was calculated using the comparative 2−ΔΔCt method and normalized to the GAPDH, ACTINB, and UBC gene levels.

**Table 1A:** Primer sequences used for the qPCR analysis.

No	Gene name	Sequence 5`- 3`
1	LAMP1 Fw	5`- CTCTAATGTCTGCAGCTCAAGG - 3`
2	LAMP1 Rev	5`- TGTACACAGCGCAGAACAGG- 3`
3	LAMP2 Fw	5`- GGCAATGATACTTGTCTGCTGGC - 3`
4	LAMP2 Rev	5`- GTAGAGCAGTGTGAGAACGGCA - 3`
5	BECLIN1 Fw	5`- CTGGACACTCAGCTCAACGTCA - 3`
6	BECLIN1 Rev	5`- CTCTAGTGCCAGCTCCTTTAGC - 3`
7	LC3B Fw	5`- GAGAAGCAGCTTCCTGTTCTGG - 3`
8	LC3B Rev	5`- GTGTCCGTTCACCAACAGGAAG - 3`
9	GAPDH Fw	5`- AGGTCCACCACTGACACGTTG - 3`
10	GAPDH Rev	5`- AGCTGAACGGGAAGCTCACT - 3`
11	ACTINB Fw	5`- AGTGTGACG TGGACATCCGGA - 3`
12	ACTINB Rev	5`- GCCAGGGCAGTGATCTCCTCCT - 3`
13	hUBC Fw	5`- TCCTGATCAGGCAGAGGTTGATCTT - 3`
14	hUBC Rev	5`- GGACCAAGTGCAGAGTGGACTCTT - 3`

### CRC patient databases and bioinformatics analysis

Statistical data processing was conducted using the Fisher-Freeman-Halton test and GraphPad Prism10. Statistical analyses of qRT-PCR and ELISA experiments were performed by a two-sided Student’s t-test. Fisher’s exact test was applied to determine the presence of nonrandom associations between clinical characteristics. All data are shown as the mean standard deviation. Considering most variables being binary or ordered categorically, we opted to use Kendall’s rank correlation analysis to investigate the patterns of association in our dataset. It allowed the generation of maximum likelihood estimates, under the assumption that the observed ordinal variables reflected continuous latent constructs. Moreover, Kendall’s tau is more accurate than Spearman’s rho with small sample sizes (33, 34). These tests were performed using the “ Kendall’s “ package in Stata v.18.

In all statistical analyses, associations were considered statistically significant at p <0.05 level (two-tailed). However, to effectively manage the rate of type I errors (false discovery rate) that may arise from multiple testing within a specific statistical test group, such as a correlation matrix consisting of numerous bivariate tests, we have employed the Benjamini-Hochberg correction method (35).

The assessment of gene expression data analysis and other statistical analyses on public datasets were performed in a multi-cohort study of public human CRC expression profiles and clinical data (n=624), based on the GEPIA 2 - Copyright ˝ 2018 Zhang’s Lab data platform.

Disease-free survival (DFS) was computed using the GEPIA2 package, where DFS was defined as the time from randomization to tumor recurrence or death and presented as Kaplan–Meier analysis.

## Results

### Clinicopathological characteristics of CRC patients

The mean age of the CRC patients was 70,8 years, with 47,4% being female. The clinicopathological characteristics of the patients are summarized in **Table 1B**. More than 70% of the CRC cases were moderately differentiated G2 (**Figure 1A**, with 61% classified as pT2 and 29% as pT3 (**Figure 1B**). Additionally, above 40% were with pN1 and pN2 lymph node invasion (**Figure 1C**). Currently, the observed survival time following the initial diagnosis ranges from 3 to 12 years (data not shown).

**Table 1B:** Demographic and clinicopathological characteristics of CRC patients.

Characteristics	Patients %	Characteristics	Patients %
Age (years), mean		Tumor stage	
Sex	70,8 ± 9,3	pT1	4
Female	47	pT2	6
Male	53	pT3	66
		pT4	24
Primary site			
Right colon	41,5	Lymphatic and vascular invasion	
Left colon	51,2	Present	48,8
Other (rectum. colon transversum)	7,3	Absent	52,2
Histology		Microsatellite instabilty	
Conventional	95,1	Present	38,7
Not determined	4,9	Absent	61,3
Histological grade (G)		Genetic mutations	
G1-G2 (low)	92	K-Ras	80,6
		N-Ras	6,5
G3 (high)	8	Other (PIK3CA/p.E545K	12,9

G1-well-differentiated tumor; G2-moderately differentiated tumor; G3-poorly differentiated tumor.

Integrated DNA Technologies, Leuven, Belgium.

### Evaluation of tumor budding

Tumor budding was classified as high (Bd2 and Bd3) in 69% (**Figure 1D**). Among these cases, half exhibited no lymph node invasion (pN0), while the remaining half were categorized as pN1 and pN2. (**Figure 1G**). Furthermore, 61% of the positive cases were moderately or poorly differentiated CRC (**Figure 1E**), while 63% were classified as pT2 and pT3 (**Figure 1F**). Absence of tumor buds (Bd0) was found in 2,4% of the patients (**Figure 1D**).

### LAMPs and autophagy in CRC and normal colon tissues

**Figure 2:**
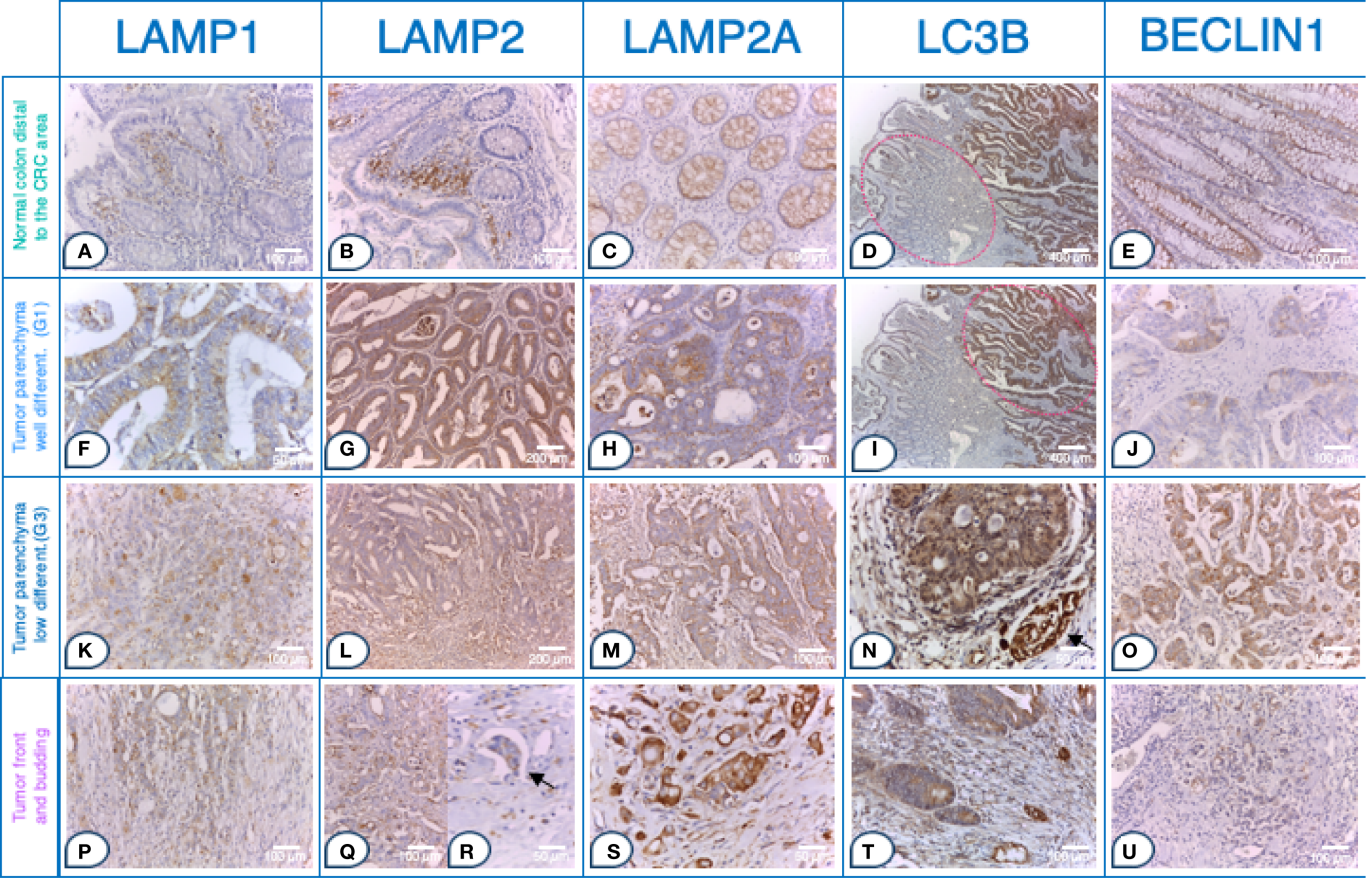
Strong LAMP and autophagy markers expression in tumor buds within the front of tumor invasion in CRC. Tissue sections. Normal colon distal to the CRC area a- e: **(A)** Glandular parenchyma 0; stroma 2+ in the upper 1/3 of the mucosal chorion. *Immunohistochemistry (IHC), anti-LAMP1*, x*200 (original magnification)*; **(B)** Glands 1 +. *IHC, anti- LAMP2*, *x200 (original magnification)*; **(C)** Expression 1 +. *IHC, anti- LAMP2A*, *x200 (original magnification)*; **(D)** Left side of the microphotograph, tumor parenchyma 1+, stroma 3+ *IHC, anti- LC3B*, *x50 (original magnification)*; **(E)** Expression 2 +. *IHC, anti-* BECLIN1, *x200 (original magnification)*; Tumor parenchyma in well-differentiated adenocarcinoma (G1) f-j: **(F)** Tumor parenchyma predominantly apical cytoplasmic signal 2+; *(IHC), anti-LAMP1*, x*400 (original magnification)*; **(G)** Tumor parenchyma 2+, stroma 2 +. *IHC, anti- LAMP2*, *x100 (original magnification)*; **(H)** In the tumor cells of tumor parenchyma 2 +. *IHC, anti- LAMP2A*, *x200 (original magnification)*; **(I)** Right side of the microphotograph, tumor parenchyma 2+, stroma 3+ *IHC, anti- LC3B*, *x200 (original magnification)*; **(J)** In the tumor cells of tumor parenchyma 2+ with expression enhanced (accentuated) around the lumen of the tumor glands. *IHC, anti-* BECLIN1, *x200 (original magnification)* Tumor parenchyma in poorly differentiated adenocarcinoma (G3) **(K–O)**: **(K)** Tumor parenchyma with marked diffuse cytoplasmic expression 3 +. *(IHC), anti-LAMP1*, x*200 (original magnification)*; **(l)** Tumor parenchyma (top) and tumor front (bottom) with clearly visible expression gradient, with more pronounced expression; expression also in the stroma around the tumor front zone in the form of vacuoles and diffusely. *IHC, anti- LAMP2*, *x100 (original magnification)*; **(M)** In the tumor cells of the tumor parenchyma 2 +. *IHC, anti- LAMP2A*, *x200 (original magnification)*; **(N)** Tumor parenchyma and stroma 3+ & internal positive normal colon with a nerve fiber 3+ (arrow). *IHC, anti- LC3B*, *x400 (original magnification)*; **(O)** 3+ expression in tumor cells from the tumor parenchyma, with expression enhanced (accentuated) around the nucleus but diffuse in nature. *IHC, anti-* BECLIN1, *x200 (original magnification)*. Tumor front and budding p-u: **(P)** Tumor front (middle) with marked diffuse cytoplasmic 3+ expression and surrounding stroma with 3 +. *(IHC), anti-LAMP1*, x*200 (original magnification)*; **(Q)** Tumor front (Budd 3) with more pronounced expression in tumor cells. *IHC, anti- LAMP2*, *x200 (original magnification)*; **(R)** Pronounced expression 3+ in tumor embolus (arrow). *IHC, anti- LAMP2*, *x400 (original magnification)*; **(S)** 3+ expression in the tumor cells of the tumor front. *IHC, anti- LAMP2A*, *x400 (original magnification)*; **(T)** Tumor front 3 +. *IHC, anti- LC3B*, *x200 (original magnification)*; **(U)** In the tumor front area of the above case **(O)**, the expression decreases; expression 1+ in the tumor front area. *IHC, anti-* BECLIN1, *x200 (original magnification)*; LAMP1, LAMP2, LAMP2A, LC3B and BECLIN1 expression levels in tumor parenchyma, stroma and front are assessed on adenocarcinomatous cells (cytokeratin 20+/cytokeratin 7-/CDX2+ markers).

The immunohistochemical analysis demonstrated higher expression of all examined molecules in CRC tissues compared to normal mucosa. Notably, there was an absence (0) or a low (1) reactivity in the normal colon glandular parenchyma and in the distal normal colon of CRC patients. **Figures 2A–E** illustrates the immunohistochemical staining of LAMP1, LAMP2, LAMP2A, LC3B and BECLIN1 in normal colon distal to the CRC area. The expression patterns in tumor parenchyma varied by differentiation status: well-differentiated adenocarcinoma (**Figures 2F–J**), poorly differentiated adenocarcinoma (**Figures 2K–O**), and tumor front and budding regions (p-t).

All LAMPs (LAMP1, LAMP2, LAMP2A) and LC3B were highly expressed in the tumor front. While in the normal colon and tumor parenchyma the expression was predominantly membranous or cytoplasmic, characterized by apical and vacuolar localization. The expression pattern in the tumor front shifted to a basal and diffuse cytoplasmic distribution, accompanied by the presence of vacuoles.

Thus, in well-differentiated adenocarcinoma (G1), the tumor parenchyma and stroma exhibited moderate to strong expression intensity (2+ to 3+) for LAMPs and LC3B (**Figures 2F–I**). In contrast, poorly differentiated CRC (G3) exhibited a distinct expression gradient, both diffusely and in vacuole-like formations, with a more pronounced signal increasing from the tumor parenchyma toward the tumor front. This expression was also apparent in the surrounding stroma near the tumor front (**Figures 2K–N**).

Strikingly, all LAMPs and LC3B were more highly expressed in the invasive tumor front. This region displayed robust immunostaining at the invasive tumor front, characterized either by marked diffuse cytoplasmic expression (3+) or by the presence of vacuoles in various regions of the cytoplasm or basally, within the tumor budding (**Figures 2P–S**). Notably, LAMP2 expression exceeded that of LAMP1 (**Figures 2G** vs. **F**, **Figures 2Q** vs. **P**) with strong LAMP2 immunostaining (3+) observed in tumor front buds and tumor emboli (**Figure 2R**).

In contrast to the other studied markers, there was a clear trend of reduced expression gradient of BECLIN1 from the tumor parenchyma to the tumor stroma and ultimately to the tumor front. In normal tissues and in poorly differentiated CRC, BECLIN1 expression was primarily localized to the paranuclear zone, which aligns with the localization of the Golgi complex, as a potential source of membrane for the generation of the phagophore (**Figures 2E, G**). In human CRC tissues, BECLIN1 was detected immunohistochemically within the plasma membrane, cytoplasm and nucleus. Poorly differentiated CRC (G3) tumor parenchyma exhibited strong (3+) expression, particularly accentuated around the nucleus but with a diffuse pattern overall (**Figure 2O**). However, BECLIN1 expression significantly decreased at the tumor front, with a low (1+) signal (**Figure 2U**).

### LAMPs and autophagy in the tumor front of CRC tissues

Next, we quantitatively compared the expression levels of LAMP1, LAMP2, LAMP2A, BECLIN1, and LC3B across different tumor regions, including tumor parenchyma, stroma, and the tumor front in adenocarcinoma cells (cytokeratin 20+/cytokeratin 7-/CDX2+).

**Figure 3:**
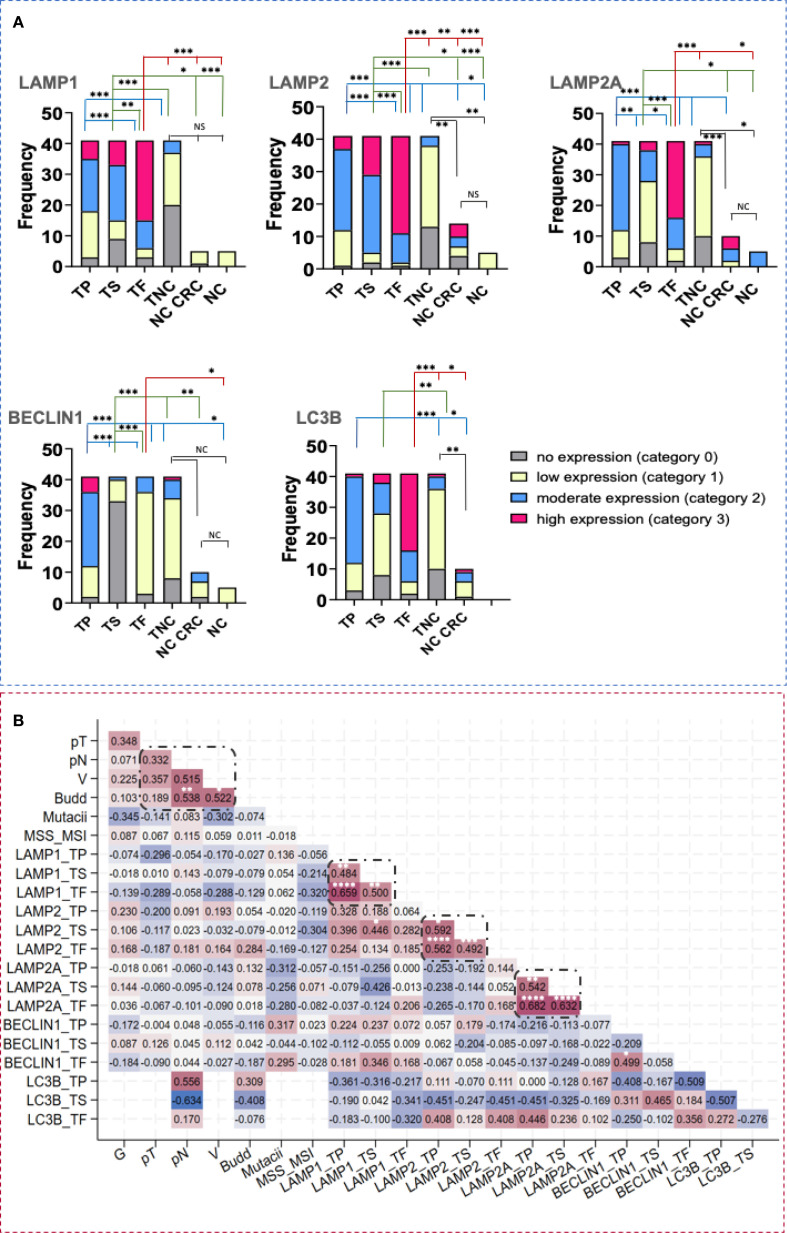
LAMP1, LAMP2, LAMP2A, BECLIN1 and LC3B tissue expression in CRC and healthy individuals. **(A)** Difference in the intensity of LAMP1, LAMP2, LAMP2A, BECLIN1 and LC3B expression in tumor parenchyma, stroma and front in CRC and in normal colon. Statistical significative differences of the protein expression levels in the tumor parenchyma and in the tumor stroma relative to the tumor front and negative controls, respectively. Expression levels were categorized into four groups (category 0- no expression, 1- low expression, 2- moderate expression, 3- high expression) in the tissue regions analyzed: (TP- tumor parenchyma; TS- tumor stroma; TF- tumor front; TNC- normal colon tissue in the CRC area; NC CRC- normal colon distal to the CRC area; NC- normal colon from nontumorous patients). *Fisher-Freeman-Halton test.* Differences are statistically significant with a Benjamini-Hochberg corrected p value (_*_ p< 0,05; _**_ p< 0,01; _***_ p< 0,001; not significant (NS), p> 0.05). For exact uncorrected and corrected p-values, see **Supplementary Table S1**, a); **(B)** Kendall`s tau correlation matrix between LAMP1, LAMP2, LAMP2A, BECLIN1 and LC3B tissue expression, budding and other clinical variables. G, histological grade; pT, tumor stage; pN, invasion in lymph nodes; L, invasion in lymphatics; V, invasion in blood vessels; MSS/MSI, microsatellite stability/microsatellite instability; TP, tumor parenchyma; TS, tumor stroma; TF, tumor front. ^*^Correlation is statistically significant with a Benjamini-Hochberg corrected p value <0.05. For exact uncorrected and corrected p-values, see **Supplementary Table S1b**); The colors span from dark blue to dark red, where dark blue denotes a r value of - 1, and dark red indicates a r value of 1.

Statistically significant differences in the expression in all examined tumor and normal colon areas were detected, with specifically intense staining observed in the front of tumor invasion relative to the tumor parenchyma and noncancerous tissue. While BECLIN1 expression was elevated in tumor tissues relative to other markers, it exhibited a reverse gradient, decreasing from the tumor parenchyma to the stroma and tumor front (**Figure 3A**).

### LAMP1, LAMP2 and BECLIN1 expression significantly correlates with budding at the tumor front in CRC

To explore potential relationship between expression levels, tumor budding and clinicopathological parameters, we conducted a Ktau analysis (**Figure 3B**). This matrix was developed by highlighting the most significant correlation coefficients, with p-values adjusted via the Benjamini-Hochberg correction. Statistically significant associations were observed among LAMP1, LAMP2, LAMP2A, and BECLIN1 across different tumor regions. Notably, we found correlations between the tumor parenchyma and stroma (r = 0.4840, p = 0.0013 for LAMP1; r = 0.5917, p = 0.0273 for LAMP2; r = 0.5418, p = 0.0014 for LAMP2A) as well as between the tumor front and both the tumor parenchyma and stroma, respectively (r = 0.6586, p = 0.0000; r = 0.4999, p = 0.0013 for LAMP1; r = 0.5622, p = 0.0000; r = 0.4917, p = 0.0007 for LAMP2; r = 0.6816, p = 0.0000; r = 0.6321, p = 0.0000 for LAMP2A). Furthermore, significant correlations were observed between the two glycoproteins, LAMP1 and LAMP2 (r= 0.446, p= 0.0297). Tumor budding was significantly associated with lymph node involvement and vascular invasion (r = 0.538, p = 0.0049; r = 0,522, p = 0.0375, respectively). A link between LAMP2 protein levels in the tumor front and the tumor budding (r= 0.2838, p = 0.0516) was also noted, though it did not remain significant after the Benjamini-Hochberg correction.

### Increased LAMP1 and decreased LAMP2 and BECLIN1 protein levels in plasma of patients with CRC

**Figure 4:**
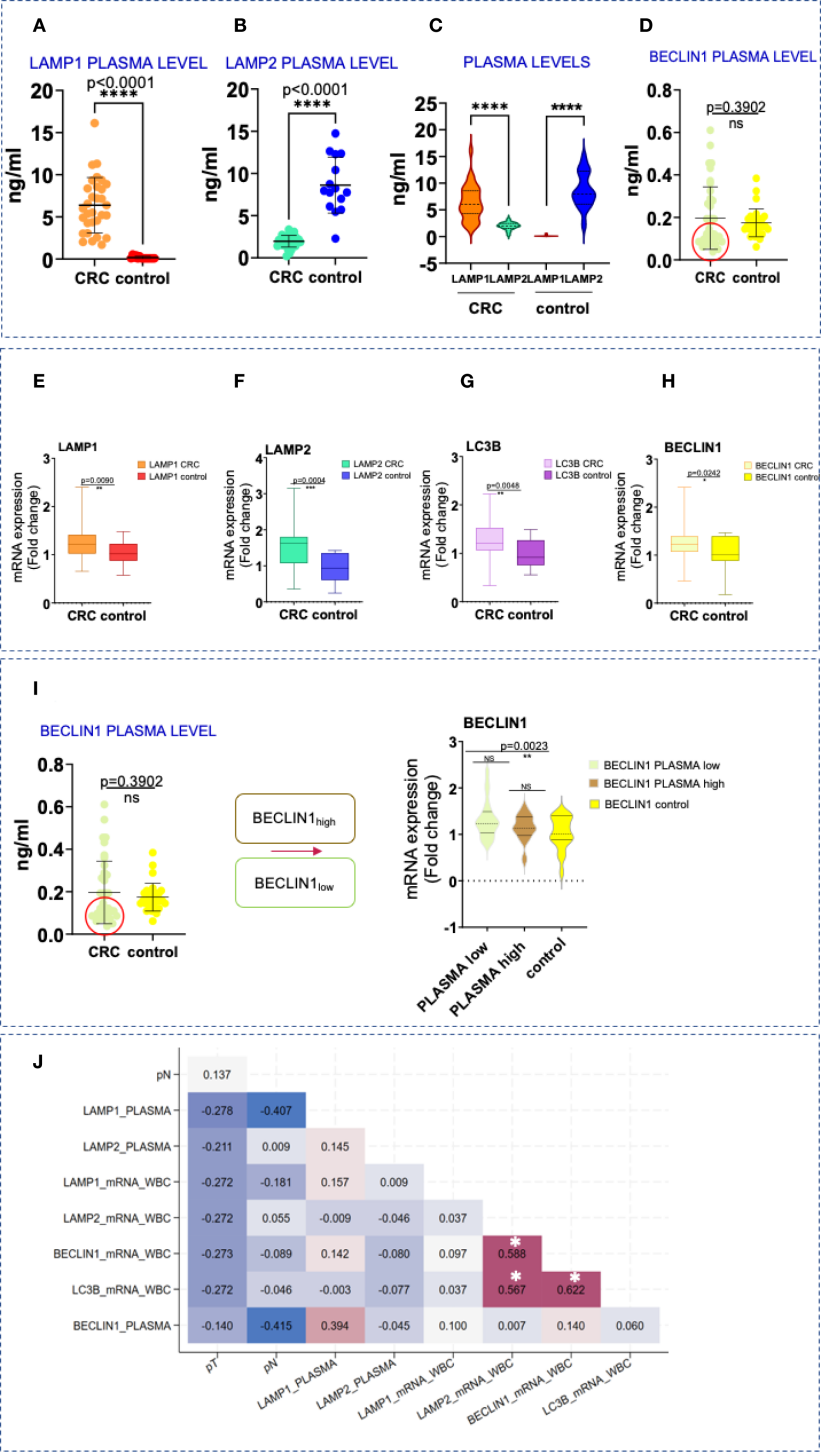
Protein and gene expression levels of LAMPs and autophagy markers in blood samples. **(A-D)** Difference in the secretory plasma circulating form of LAMP1, LAMP2, and BECLIN1 in CRC and in healthy patients. Statistical significative differences of the protein plasma levels in the CRC blood samples relative to the healthy controls, respectively. Statistically significative differences were calculated by *t-test* with Welch`s correction, ****p<0,0001; not significant (ns), p> 0.05. **(E–H)** LAMP1, LAMP2, BECLIN1 and LC3B overexpression in CRC. qPCR analysis of LAMP1, LAMP2, BECLIN1 and LC3B mRNA levels in WBC in CRC and healthy donors. Data are shown as mean ± SD. Statistically significative differences were calculated by *t-test.* p value (*p< 0,05; **p<0,001; ***p< 0,0001; ****p<0,0001). **(I)** Тwo subcategories, with lower levels of BECLIN1 (within the red circle) and higher- above. BECLIN1_low_ plasma concentrations are associated with CRC patients displaying elevated transcript levels in WBCs (the figure on the right- *t-test* with Welch`s correction, box plot analysis). **(J)** Kendall`s tau rank correlation matrix between LAMP1, LAMP2, BECLIN1 and LC3B plasma protein and gene expression levels in WBC and other clinical variables. pT, tumor stage; pN, invasion in lymph nodes; ^*^Correlation is statistically significant with a Benjamini-Hochberg corrected p value < 0.05. For exact uncorrected and corrected p-values, see **Supplementary Table S1c**); The colors span from dark blue to dark red, where dark blue denotes a r value of - 1, and dark red indicates a r value of 1.

In addition to evaluating the tissue and spatial expression of lysosomal and autophagy-related molecules, we assessed the protein levels of LAMP1 and LAMP2, along with BECLIN1, in plasma samples. Our analysis revealed a significant increase in LAMP1 concentrations compared to the control group (**Figure 4A**). Interestingly, we observed a decrease in the secretory form of LAMP2 in CRC patients when compared to healthy individuals (**Figure 4B**). Notably, in CRC patients, higher levels of LAMP1 were associated with lower levels of LAMP2, while the opposite was true in the control group (**Figure 4C**). Although overall BECLIN1 plasma levels did not differ significantly between both groups, two distinct CRC subgroups with BECLIN _high_ and BECLIN1_low_ expression were identified (**Figure 4D**).

### Upregulated LAMPs and autophagy genes in CRC patients and healthy controls

Gene expression analysis in WBCs from CRC patients revealed significant upregulation of LAMP1, LAMP2, BECLIN1 and LC3B compared to controls (**Figures 4E–H**). These findings were also confirmed by the immunohistochemical analysis shown in **Figure 2**. While a similar trend was detected between the expression levels of LAMP1 and LAMP2,moderate to strong correlations were registered between LAMP2, BECLIN1 and LC3B, respectively (r=0.588, p< 0.001 for LAMP2 and BECLIN1; r=0.567, p< 0.001 for LAMP2 and LC3B; r=0.622, p< 0.001 for BECLIN1 and LC3B) (**Figure 4G**).

Interestingly, CRC patients with low plasma BECLIN1 levels exhibited higher transcript levels in WBCs (**Figure 4I**), a pattern also noted for LAMP2.

### Prognostic significance of LAMP1 and LAMP2 in CRC

**Figure 5:**
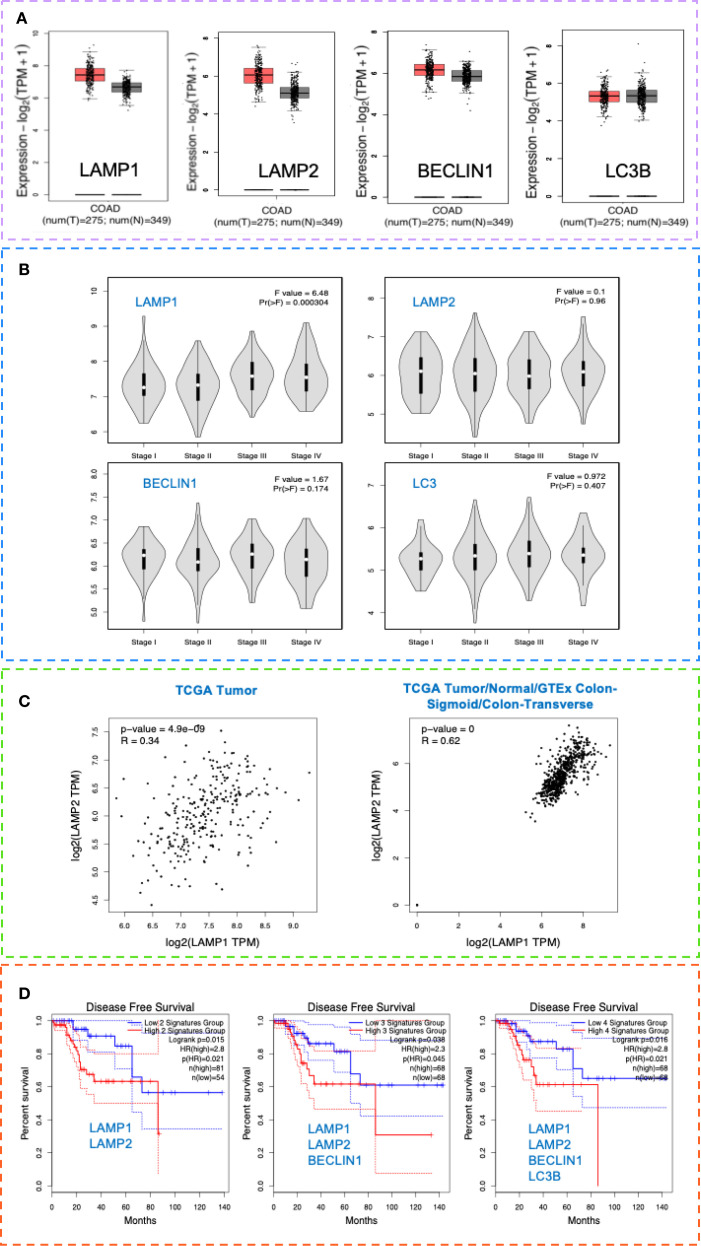
LAMP and autophagy signatures as potential markers of invasiveness in CRC and their prognostic significance. **(A)** LAMP1, LAMP2, BECLIN1 and LC3B tissue expression in CRC and healthy individuals. Box plot analysis of LAMP1, LAMP2, BECLIN1 and LC3B mRNA levels in tissues in CRC TCGA COAD tumor and TCGA COAD normal and GTEx normal data, using the GEPIA 2 data platform-Copyright ^©^ 2018 Zhang’s Lab. For the gene expression profile were considered: Differential Method ANOVA; ILog2FCI Cutoff 1, q-value Cutoff: 0.0. **(B)** CRC stage distribution (I-IV) of LAMP1, LAMP2, BECLIN1 and LC3B in the TCGA data COAD tumor, using the GEPIA 2 data platform - Copyright ^©^ 2018 Zhang’s Lab. **(C)** Correlation analysis of LAMP1 and LAMP2 gene signatures in the TCGA data COAD tumor (left, r=0.34, p=4.9e-09) and in the TCGA Tumor/Normal and the GTEx data for the colon-sigmoid/transverse sections (right, r=0.62, p=0), using the GEPIA 2 data platform - Copyright ^©^ 2018 Zhang’s Lab. Pearson correlation coefficient was calculated using the expression data sets derived from TCGA COAD tumor (n=275), TCGA COAD normal (n=41), as well as GTEx colon sigmoid and colon transverse (n=308). **(D)** Cancer-specific survival analysis in the context of LAMP1, LAMP2, BECLIN1 and LC3B expression in patients with CRC (TCGA). Kaplan-Meier analysis was performed, comparing TCGA-COAD patients with high 75% Cutoff and patients with low 25% Cutoff expression values within 140 months, using the GEPIA 2 data platform - Copyright ^©^ 2018 Zhang’s Lab, where MSI-H (n=52), MSI-L (n=52) and MSS (n=184).

Public data analysis (GEPIA 2 - Copyright ^©^ 2018 Zhang’s Lab) confirmed that LAMP1, LAMP2, BECLIN1, and LC3B were significantly upregulated in CRC tissues (**Figure 5A**), particularly in later disease stages (**Figure 5B**). A good correlation was observed in the expression of LAMP1 and LAMP2 in the TCGA data (r=0.34, p=4.9e-09). Moreover, a strong correlation was noted when analyzing TCGA Tumor/Normal and the GTEx data for the colon-sigmoid/transverse sections (r=0.62, p=0) (**Figure 5C**).

In addition, we also followed the prognostic significance of LAMP1 and LAMP2. Based on the expression levels, a Kaplan-Meier analysis was performed, comparing patients with high and low expression values. As presented in **Figure 5D**, the high expression of both signatures determined a shorter disease-free survival (DFS) in the TCGA cohort compared to patients with low expression. Notably, increased BECLIN1 expression improved survival by up to 30% in the LAMP1_high_LAMP2_high_ group, whereas concurrent upregulation of all four markers resulted in a DFS rate of 0% after 85 months. Collectively, these findings indicate that the upregulation of the examined proteins correlates with poor prognosis.

## Discussion

To the best of our knowledge, the present study constitutes the first comprehensive examination of LAMP1 and LAMP2 expression across distinct regions of CRC tissue. Initially, we explored the clinical significance of lysosomal and autophagy signatures in relation to conjunction with tumor budding and the clinicopathological characteristics in CRC to better understand their prognostic potential. Our findings reveal a clear upregulation of LAMP1, LAMP2 and LAMP2A in the tumor parenchyma, stroma and front of CRC tissues. These observations were further corroborated by comparative gene expression analysis using publicly available datasets. Furthermore, upregulation of autophagy-associated molecules, such as BECLIN1 and LC3B, was also observed in the CRC study group and confirmed by the data analyzed from public sources.

Our study indicates that LAMP1 and LAMP2 are not only expressed in tumor cells but are also prominently present at the tumor front - both in tumor and stromal cells. A key finding is their specific localization reported in the tumor buds, which suggests a role in the invasive potential of cancer cells. In support of this, previous research by Sitoh et al. reported elevated expression of LAMP1 and LAMP2 on the surface of highly metastatic cells (mCRC), which the authors linked to tumor progression and the acquisition of metastatic traits (36). Additional studies have highlighted dysregulated expression of other LAMP molecules, specifically LAMP3 (37), LAMP4 (38) and LAMP5, whose upregulation has been associated with CRC invasion and metastasis, serving as poor prognostic biomarkers (39).

Additionally, the fact that we observed overexpressed levels of LAMP2A at the tumor stroma and front, suggests that CMA may be active at these sites in our CRC study group. Notably, a 2.8-fold upregulation of CMA is recorded in multiple cancer cell types (40, 41) which fosters cancer cells’ growth and development (42, 43). Supporting this, Peng et al. reported a significant increase in LAMP2A expression in CRC patients and mouse models (44), while the inhibition of CMA through the downregulation of LAMP2A resulted in inhibited CT26 colon cell proliferation and a decrease in lung tumor burden in mice (43).

The spatial expression pattern of LC3B in our study closely mirrored that of LAMP1, LAMP2 and LAMP2A, with marked enrichment at the tumor front within the tumor buds. Few studies in the last years showed conflictive results associated with varied LC3B levels. In CRC, the overexpression of LC3B is positively correlated with lymph node metastasis, a factor typically associated with poor prognosis. Conversely, elevated LC3B is also linked to improved overall survival outcomes (45). A confirmatory study in CRC demonstrated that low cytoplasmic staining for LC3B and p62 is associated with worse overall survival, while the high expression of both markers, is indicative of impaired autophagic activation, and correlated with better outcomes (46).

There is evidence that BECLIN1 plays a significant role in the growth and metastasis of CRC. Patients exhibiting extensive overexpression or underexpression of BECLIN1 had a markedly poorer overall survival rate (47). We detected moderately increased levels of BECLIN1 in tumor parenchyma, which aligns with the expression patterns of the other examined markers. Conversely, minimal expression was observed in the tumor stroma, while the immunostaining at the tumor front was comparable to that in normal colon tissue. The reasons for such a reduction remain unclear. A possible explanation is that its specific activity may not be required at the site of tumor invasion because BECLIN1 is essential for autophagy induction by initiating phagophore formation at the ER, mitochondria, or ER–plasma membrane contact sites. The progression of CRC might not require further BECLIN1 activity as decreased BECLIN1 has been associated with CRC invasion (48). Our data confirms that this molecule is primarily concentrated around the nucleus, specifically within the tumor parenchyma of the CRC studied group and is reduced in the invasive front. In poorly differentiated (G3) CRC a 3+ expression was noted. However, in our CRC study group, only 9.7% of cases were classified as G3, whereas 68% were G2.

Furthermore, moderate correlations were found between the tissue expression of LAMP1 and LAMP2, as well as between BECLIN1 in the tumor front and LAMP1 in the tumor stroma. Notably, the assessment of tissue samples indicated an upward trend in the expression levels of LAMP1, LAMP2, LAMP2A, BECLIN1, and LC3B correlating with more advanced stages of CRC, especially considering that 90% of our study cohort was classified as pT3-pT4. This trend was consistent with gene expression data from publicly available databases.

Currently, there is a lack of research concerning the expression of LAMPs and tumor budding in CRC. Although a notably enhanced staining for LAMP1 and LAMP2, together with LC3B was observed within the tumor buds, no significant correlation was detected between their expression levels and the budding score. Nonetheless, association and relationship were identified between budding and the invasion in lymphatics and blood vessels. Therefore, this trend suggests a potential role of these glycoproteins in CRC invasiveness.

Given the emerging role of liquid biopsy in CRC detection, molecular profiling and treatment response prediction, we also assessed the protein and gene expression levels of the lysosomal and autophagy-associated markers in blood samples. To our knowledge, this is the first research reporting the transcriptional and protein levels of lysosomal and autophagy-related molecules in WBCs and plasma in the context of CRC. Notably, transcripts for all examined molecules were significantly overexpressed in the CRC group compared to healthy individuals (**Figures 4E–H**), with strong correlation observed between LAMP2, BECLIN1 and LC3B (**Figure 4J**). However, while plasma protein levels of LAMP1 remained elevated in the CRC patients, LAMP2 and BECLIN1_low_ protein levels were paradoxically higher in the control group. The regulatory mechanism responsible for the reduction of the circulating LAMP2 and BECLIN1_low_ forms requires further exploration and analysis. Post-translational modifications, mRNA stability, protein degradation, secretion, immune-mediated clearance and altered glycosylation are all potential factors for consideration. E3 ubiquitin ligases play a critical role in colon carcinogenesis by regulating protein ubiquitination and proteasomal degradation, thereby affecting key signaling (49). The tripartite motif (TRIM) family, which includes RING-finger E3 ligases, has been shown to regulate autophagy and protein degradation. For example, TRIM3 promotes BECLIN1 degradation in Ewing sarcoma cells (50). Changes in secretion pathways can also impact protein levels. LAMP2 is commonly found on exosomes from immune cells, but the specific secretory mechanisms involved remain unclear (51). The trafficking of LAMP1 is still debated, with evidence suggesting its endocytosis relies on clathrin, dynamin, and AP2 (52, 53). LAMP1 and LAMP2 are both heavily glycosylated, though LAMP1 undergoes additional modification by fucosyltransferases, which may affect their secretion. BECLIN1 also contributes to the secretory pathway through its non-autophagy functions, particularly via the PI3K-III complex. BECLIN1 levels can be altered by protein interactions, proteolytic cleavage, or epigenetic regulation, and reduced levels of BECLIN1 or its partners VPS34 and UVRAG can impair TGF-β signaling (54). Immune-mediated clearance is another important mechanism. Recent studies have shown that tumor-derived exosomes can impair T-cell function and promote Th17 cell differentiation (55), influencing CRC progression (56). STAT3 may directly affect lysosomal composition and function, with LAMP2 as a key target (57). Overall, these findings suggest that distinct and multiple mechanisms may modulate plasma protein levels of LAMP2 and BECLIN1. Further research is needed to determine whether the reduced plasma levels of LAMP2 and BECLIN1 observed in our CRC study group have pro- or anti-tumor effects. However, these aspects were not the primary focus of our study. Whether the reduced plasma levels of LAMP2 and BECLIN1 observed in our CRC study group exert pro- or anti-tumor effects definitely need more investigation. Whether the downregulated levels are required for the invasion and survival of circulating tumor cells, as well as their potential association with an altered and more metastatic phenotype, necessitates further analysis. It is essential to emphasize that the autophagic flux present within the plasma is distinct from the autophagy occurring in tumor cells and may play a significant role in regulating tumor cell escape. In this regard, work by Han and team has shown that impaired macroautophagy is required for the CMA-induced growth and metastasis in human breast cancer cells (58). Data presented by Hu et al. indicate that BECLIN1 deficiency in CRC samples has no effect on cancer cell growth but significantly increases their mobility and invasion (48). Additionally, an interesting correlation was uncovered between LAMP1 levels measured in plasma and transcript levels in WBCs of CRC cases (**Figure 4J**). These findings all together indicate that not only tissue but also blood (plasma and WBCs) levels of these glycoproteins could serve as valuable biomarkers.

To date, no prior studies have investigated the expression of LAMPs in relation to tumor budding and autophagy in CRC. Therefore, we are the first to demonstrate moderate to strong expression of LAMP1, LAMP2 and LAMP2A in the tumor stroma and front in CRC tissues. The significant association between the expression of these proteins and the tumor budding indicates their implication in cancer cells’ invasiveness. Given the higher protein and gene expression levels of LAMP1 and LAMP2 in CRC tissues, as evidenced by both our findings and public data, we explored the prognostic significance of LAMP1 and LAMP2, individually and in combination with BECLIN1 and LC3B. We found that patients with high LAMP1 and LAMP2 experienced shorter DFS compared to those with low expression levels within the TCGA cohort. Notably, high BECLIN1 expression improved survival in the same study group. Our findings are consistent with previous investigations that have established a positive correlation between BECLIN1 expression and favorable prognosis in patients with intestinal-type carcinoma (59) and specifically in stage IIIB CRC (60). Additionally, BECLIN1 overexpression *in vivo* significantly inhibits the proliferation of CRC cells in xenograft models, inducing apoptosis through the upregulation of LC3B (61). However, we discovered that the elevated expression of all four markers resulted in a significantly reduced DFS after 85 months. Collectively, these findings indicate that the upregulation of these proteins correlates with poor prognosis.

Our results demonstrate that all the three major forms of autophagy might be activated in our CRC group. The trafficking of lysosomes toward the tumor stroma plays a crucial role in remodeling the EMT, thereby promoting invasion at the tumor front. Notably, numerous studies indicated that repositioning of lysosomes toward the cell periphery is essential for lysosomal exocytosis (62) with LAMP1 being crucial for the docking phase at the plasma membrane during this process (63). Furthermore, recent research showed that lysosomal exocytosis is fundamental in remodeling the extracellular matrix and facilitating EMT activation. Thus, cancer cells exploit the mechanism of lysosomal exocytosis to acidify the tumor microenvironment, restructure the extracellular matrix, and communicate with surrounding cells in the tumor stroma. Additionally, we also observed elevated LAMP2A at the tumor stroma and front. All these facts suggest that, alongside CMA, microautophagy is possibly taking place at the invasive tumor front. Furthermore, the prominent staining of LC3B that we observed at the tumor front underscores the active role of macroautophagy in this region. It is considered as essential for the subsequent fusion of autophagosomes with lysosomes. Based on our data, we hypothesize that while BECLIN1 is essential for the initial stages of macroautophagy, CMA and potentially microautophagy may also occur. Thus, the evidence presented by us and others indicate that all three forms of autophagy create conducive conditions for CRC progression and invasion (64). The complex molecular networks that underlie the distinct autophagic pathways, in the context of cancerogenesis, and in particular the double-edge interplay between autophagy and cancer and CRC have been a subject of extensive investigation during the last years. Autophagy often plays a suppressive role during the initial stages of carcinogenicity, while in the later stages of cancer development, it can play a promoting role. It is extremely important to determine the regulation of this duality of autophagy in the development of CRC and to identify the molecules involved, as well as the signals and the mechanisms behind.

In addition to autophagy, researchers have increasingly focused on tumor budding as a potential marker for EMT, given that tumor buds communicate with multiple components of the tumor stroma (65). Notably, in pT1 CRC, tumor budding serves as a strong predictor of lymph node metastasis (66) and is associated with poor prognosis in stage II CRC (67). It is crucial to continue to delve into the intricacies of tumor budding and autophagy machinery to enhance our understanding of cancer prognosis and ultimately improve patient outcomes. Our results also suggest that LAMP1, LAMP2 and LC3B may play a significant role in cancer progression, as evidenced by their abundant expression at the tumor front and the observed correlation between tumor budding and lymphatic and blood vessel invasion. The spatial implications of the budding phenomenon and the potential of LAMPs and autophagy molecules as biomarkers for the diagnosis and stratification of CRC warrant further investigation. The standard treatment protocol for CRC patients typically involves oxaliplatin and 5-fluorouracil (68, 69). However, autophagy induced by genomic and epigenetic modifications has been linked to resistance to these chemotherapeutic agents. Recent studies have established a link between CMA and resistance to antitumor therapies (70) identifying LAMP2A as a mediator of cisplatin resistance in CRC (9). Additionally, increased LAMP1 expression has been observed in breast cancer cells following the development of doxorubicin resistance (71). Inhibition of LAMP2 has been shown to reverse macrophage activation and enhance tumor cytotoxicity. Suppression of autophagy may therefore improve tumor cell responsiveness to standard therapies and help overcome chemoresistance. Down-regulation of LAMP proteins increases drug sensitivity in human colon and breast carcinoma cells. O’Donovan et al. demonstrated that lithium treatment sensitizes CRC cells to cisplatin or 5-FU and depletes LAMP1, LAMP2, and cathepsin B, indicating compromised lysosomal stability (72). Furthermore, lithium combined with oxaliplatin synergistically inhibits tumor growth in a xenograft colorectal carcinoma model (73). Further investigation is required to elucidate the specific mechanisms of LAMPs in order to develop more effective therapeutic strategies, particularly in the context of personalized and combination therapies, with the aim of improving survival rates and quality of life for cancer patients.

However, our study has its limitations. The analysis sample is modest, which could explain some of the null associations observed. Future validation studies are needed to test the robustness of our findings. The limited sample size resulted in insufficient statistical power for exploring the potential effect modifiers at different stages of CRC progression (as stage II vs III/IV). However, since CRC is a highly heterogeneous disease stratified analyses could have revealed associations that may have been obscured in the total sample group. Another limitation is that the study is exploratory and correlational, providing only suggestive evidence of causality. Actual experimental manipulation in CRC models would provide stronger mechanistic evidence.

In summary, we present novel data on the expression of LAMP1, LAMP2 and LAMP2A, together with autophagy-associated BECLIN1 and LC3B glycoproteins in parallel with tumor budding in CRC. Our findings indicate that the lysosomal markers together with LC3B accumulated at the tumor front may suggest a role in tumor aggressiveness. This research offers a new perspective on the clinical relevance of the identified signatures and the spatial immunochemical expression within the tumor buds. These findings contribute to our understanding of CRC progression and may have implications for the development of targeted therapeutic strategies.

## Data Availability

All data supporting the findings of the study are available within the paper and **Supplementary Files**.

## References

[1] Morgan E Arnold M Gini A Lorenzoni V Cabasag CJ Laversanne M. Global burden of colorectal cancer in 2020 and 2040: incidence and mortality estimates from GLOBOCAN. Gut. (2023) 72:338. doi: 10.1136/gutjnl-2022-327736, PMID: 36604116

[2] Mauri G Vitiello PP Sogari A Crisafulli G Sartore-Bianchi A Marsoni S. Liquid biopsies to monitor and direct cancer treatment in colorectal cancer. Br J Cancer. (2022) 127:394–407. doi: 10.1038/s41416-022-01769-8, PMID: 35264786 PMC9346106

[3] Zlobec I Berger MD Lugli A. Tumour budding and its clinical implications in gastrointestinal cancers. Br J Cancer. (2020) 123:700–8. doi: 10.1038/s41416-020-0954-z, PMID: 32601463 PMC7462864

[4] Eskelinen EL. Autophagy: Supporting cellular and organismal homeostasis by self-eating. Int J Biochem Cell Biol. (2019) 111:1–10. doi: 10.1016/j.biocel.2019.03.010, PMID: 30940605

[5] White E DiPaola RS. The double-edged sword of autophagy modulation in cancer. Clin Cancer Res. (2009) 15:5308–16. doi: 10.1158/1078-0432.CCR-07-5023, PMID: 19706824 PMC2737083

[6] Mathew R Kongara S Beaudoin B Karp CM Bray K Degenhardt K. Autophagy suppresses tumor progression by limiting chromosomal instability. Genes Dev. (2007) 21:1367–81. doi: 10.1101/gad.1545107, PMID: 17510285 PMC1877749

[7] Mathew R Karp CM Beaudoin B Vuong N Chen G Chen HY. Autophagy Suppresses Tumorigenesis through Elimination of p62. Cell. (2009) 137:1062–75. doi: 10.1016/j.cell.2009.03.048, PMID: 19524509 PMC2802318

[8] Yun CW Lee SH. The roles of autophagy in cancer. Int J Mol Sci. (2018) 19:3466. doi: 10.3390/ijms19113466, PMID: 30400561 PMC6274804

[9] Li J Hou N Faried A Tsutsumi S Kuwano H. Inhibition of autophagy augments 5-fluorouracil chemotherapy in human colon cancer *in vitro* and *in vivo* model. Eur J Cancer. (2010) 46:1900–9. doi: 10.1016/j.ejca.2010.02.021, PMID: 20231086

[10] Li YJ Lei YH Yao N Wang CR Hu N Ye WC. Autophagy and multidrug resistance in cancer. Chin J cancer. (2017) 36:52. doi: 10.1186/s40880-017-0219-2, PMID: 28646911 PMC5482965

[11] Sarafian V Jadot M Foidart JM Letesson JJ Van den Brûle F Castronovo V. Expression of Lamp-1 and Lamp-2 and their interactions with galectin-3 in human tumor cells. Int J Cancer. (1998) 75:105–11. doi: 10.1002/(SICI)1097-0215(19980105)75:1<105::AID-IJC16>3.0.CO;2-F, PMID: 9426697

[12] Li L Xu Y Wang W Zhang G Ma M Huang J. LAMP1 is more sensitive than LAMP2 in predicting prognosis of esophageal squamous cell carcinoma. (2020) 9:2243–8. doi: 10.21037/tcr.2020.03.27, PMID: 35117584 PMC8798246

[13] Sarafian VS Marinova TT Gulubova MV. Differential expression of LAMPs and ubiquitin in human thymus. APMIS. (2009) 117:248–52. doi: 10.1111/j.1600-0463.2009.02437.x, PMID: 19343823

[14] Sarafian VS Dikov DI. LAMPs and ABH histo-blood group antigens in granulation tissue. APMIS. (2007) 115:701–6. doi: 10.1111/j.1600-0463.2007.apm_576.x, PMID: 17550377

[15] Eskelinen EL. Roles of LAMP-1 and LAMP-2 in lysosome biogenesis and autophagy. Mol Aspects Med. (2006) 27:495–502. doi: 10.1016/j.mam.2006.08.005, PMID: 16973206

[16] Liu X Liao X Rao X Wang B Zhang J Xu G. The lysosomal membrane protein LAMP-2 is dispensable for PINK1/Parkin-mediated mitophagy. FEBS Lett. (2020) 594:823–40. doi: 10.1002/1873-3468.13663, PMID: 31693752

[17] Pajares M Rojo AI Arias E Díaz-Carretero A Cuervo AM Cuadrado A. Transcription factor NFE2L2/NRF2 modulates chaperone-mediated autophagy through the regulation of LAMP2A. Autophagy. (2018) 14:1310–22. doi: 10.1080/15548627.2018.1474992, PMID: 29950142 PMC6103698

[18] Cuervo AM Dice JF. Unique properties of lamp2a compared to other lamp2 isoforms. J Cell Sci. (2000) 113:4441–50. doi: 10.1242/jcs.113.24.4441, PMID: 11082038

[19] Qiao L Hu J Qiu X Wang C Peng J Zhang C. LAMP2A, LAMP2B and LAMP2C: similar structures, divergent roles. Autophagy Taylor Francis Ltd. (2023) 19:2837–52. doi: 10.1080/15548627.2023.2235196, PMID: 37469132 PMC10549195

[20] Chi C Leonard A Knight WE Beussman KM Zhao Y Cao Y. LAMP-2B regulates human cardiomyocyte function by mediating autophagosome–lysosome fusion. Proc Natl Acad Sci U S A. (2019) 116:556–65. doi: 10.1073/pnas.1808618116, PMID: 30584088 PMC6329949

[21] Fujiwara Y Furuta A Kikuchi H Aizawa S Hatanaka Y Konya C. Discovery of a novel type of autophagy targeting RNA. Autophagy. (2013) 9:403–9. doi: 10.4161/auto.23002, PMID: 23291500 PMC3590259

[22] Fujiwara Y Kikuchi H Aizawa S Furuta A Hatanaka Y Konya C. Direct uptake and degradation of DNA by lysosomes. Autophagy. (2013) 9:1167–71. doi: 10.4161/auto.24880, PMID: 23839276 PMC3748189

[23] Hase K Fujiwara Y Kikuchi H Aizawa S Hakuno F Takahashi SI. RNautophagy/DNautophagy possesses selectivity for RNA/DNA substrates. Nucleic Acids Res. (2015) 43:6439–49. doi: 10.1093/nar/gkv579, PMID: 26038313 PMC4513860

[24] Zheng Hc Zhao S Xue H Zhao Eh Jiang Hm Hao Cl. The roles of beclin 1 expression in gastric cancer: A marker for carcinogenesis, aggressive behaviors and favorable prognosis, and a target of gene therapy. Front Oncol. (2020) 10. doi: 10.3389/fonc.2020.613679, PMID: 33425768 PMC7787063

[25] Bednarczyk M Fatyga E Dzięgielewska-Gęsiak S Waniczek D Grabarek B Zmarzły N. The expression patterns of BECN1, LAMP2, and PINK1 genes in colorectal cancer are potentially regulated by micrornas and CpG islands: An in silico study. J Clin Med. (2020) 9:1–10. doi: 10.3390/jcm9124020, PMID: 33322704 PMC7764710

[26] Baskaran S Carlson LA Stjepanovic G Young LN Kim DJ Grob P. Architecture and dynamics of the autophagic phosphatidylinositol 3-kinase complex. Elife. (2014) 3:e05115. doi: 10.7554/eLife.05115, PMID: 25490155 PMC4281882

[27] Kabeya Y Mizushima N Ueno T Yamamoto A Kirisako T Noda T. LC3, a mammalian homologue of yeast APG8P, is localized in autophagosome membranes after processing. EMBO J. (2000) 19:5720–8. doi: 10.1093/emboj/19.21.5720, PMID: 11060023 PMC305793

[28] Ghavami S Shojaei S Yeganeh B Ande SR Jangamreddy JR Mehrpour M. Autophagy and apoptosis dysfunction in neurodegenerative disorders. Prog Neurobiol. (2014) 112:24–49. doi: 10.1016/j.pneurobio.2013.10.004, PMID: 24211851

[29] Zhao H Yang M Zhao B. Beclin 1 and LC3 as predictive biomarkers for metastatic colorectal carcinoma. (2017) 8:59058–67. doi: 10.18632/oncotarget.19939, PMID: 28938618 PMC5601714

[30] Geng B Pan J Zhao T Ji J Zhang C Che Y. Chitinase 3-like 1-CD44 interaction promotes metastasis and epithelial-to-mesenchymal transition through β-catenin/Erk/Akt signaling in gastric cancer. J Exp Clin Cancer Res. (2018) 37:208. doi: 10.1186/s13046-018-0876-2, PMID: 30165890 PMC6117920

[31] Kazakova M Ivanova T Dikov D Molander D Simitchiev K Sbirkov Y. Strong YKL-40 expression in the invasive tumor front of colorectal cancer–A pilot study. Heliyon. (2024) 10:e27570. doi: 10.1016/j.heliyon.2024.e27570, PMID: 38495157 PMC10940939

[32] Lugli A Karamitopoulou E Zlobec I. Tumour budding: a promising parameter in colorectal cancer. Br J Cancer. (2012) 106:1713–7. doi: 10.1038/bjc.2012.127, PMID: 22531633 PMC3364122

[33] Fieller EC Hartley HO Pearson ES. Tests for rank correlation coefficients. I. Biometrika. (1957) 44:470–81. doi: 10.1093/biomet/44.3-4.470

[34] Arndt S Turvey C Andreasen NC. Correlating and predicting psychiatric symptom ratings: Spearmans r versus Kendalls tau correlation. J Psychiatr Res. (1999) 33:97–104. doi: 10.1016/S0022-3956(98)90046-2, PMID: 10221741

[35] Benjamini Y Hochberg Y. Controlling the false discovery rate: A practical and powerful approach to multiple testing. J R Stat Society: Ser B (Methodological). (1995) 57:289–300. doi: 10.1111/j.2517-6161.1995.tb02031.x

[36] Saitoh O Wang WC Lotan R Fukuda M. Differential glycosylation and cell surface expression of lysosomal membrane glycoproteins in sublines of a human colon cancer exhibiting distinct metastatic potentials. J Biol Chem. (1992) 267:5700–11. doi: 10.1016/S0021-9258(18)42823-2, PMID: 1544942

[37] Zhu J Long T Gao L Zhong Y Wang P Wang X. RPL21 interacts with LAMP3 to promote colorectal cancer invasion and metastasis by regulating focal adhesion formation. Cell Mol Biol Lett. (2023) 28:31. doi: 10.1186/s11658-023-00443-y, PMID: 37062845 PMC10108486

[38] Zhang J Li S Liu F Yang K. Role of CD68 in tumor immunity and prognosis prediction in pan-cancer. Sci Rep. (2022) 12:7844. doi: 10.1038/s41598-022-11503-2, PMID: 35550532 PMC9098459

[39] Martinez-Romero J Bueno-Fortes S Martín-Merino M Ramirez de Molina A De Las Rivas J. Survival marker genes of colorectal cancer derived from consistent transcriptomic profiling. BMC Genomics. (2018) 19:857. doi: 10.1186/s12864-018-5193-9, PMID: 30537927 PMC6288855

[40] Krause GJ Kirchner P Stiller B Morozova K Diaz A Chen KH. Molecular determinants of the crosstalk between endosomal microautophagy and chaperone-mediated autophagy. Cell Rep. (2023) 42:113529. doi: 10.1016/j.celrep.2023.113529, PMID: 38060380 PMC10807933

[41] Rios J Sequeida A Albornoz A Budini M. Chaperone mediated autophagy substrates and components in cancer. Front Oncol. (2021) 10. doi: 10.3389/fonc.2020.614677, PMID: 33643916 PMC7908825

[42] Ding ZB Fu XT Shi YH Zhou J Peng YF Liu WR. Lamp2a is required for tumor growth and promotes tumor recurrence of hepatocellular carcinoma. Int J Oncol. (2016) 49:2367–76. doi: 10.3892/ijo.2016.3754, PMID: 27840904

[43] Kon M Kiffin R Koga H Chapochnick J Macian F Varticovski L. Chaperone-mediated autophagy is required for tumor growth. Sci Transl Med. (2011) 3:109ra117. doi: 10.1126/scitranslmed.3003182, PMID: 22089453 PMC4000261

[44] Peng Jq Han Sm Chen Zh Yang J Pei Yq Bao C. Chaperone-mediated autophagy regulates apoptosis and the proliferation of colon carcinoma cells. Biochem Biophys Res Commun. (2020) 522:348–54. doi: 10.1016/j.bbrc.2019.11.081, PMID: 31761324

[45] Shen N Wang L Wu J Chen X Hu F Su Y. Meta-;analysis of the autophagy-;associated protein LC3 as a prognostic marker in colorectal cancer. Exp Ther Med. (2023) 26:492. doi: 10.3892/etm.2023.12191, PMID: 37753301 PMC10518644

[46] Niklaus M Adams O Berezowska S Zlobec I Graber F Slotta-Huspenina J. Expression analysis of LC3B and p62 indicates intact activated autophagy is associated with an unfavorable prognosis in colon cancer. (2017) 8:54604–54615. doi: 10.18632/oncotarget.17554, PMID: 28903368 PMC5589607

[47] Koukourakis MI Giatromanolaki A Sivridis E Pitiakoudis M Gatter KC Harris AL. Beclin 1 over- and underexpression in colorectal cancer: distinct patterns relate to prognosis and tumour hypoxia. Br J Cancer. (2010) 103:1209–14. doi: 10.1038/sj.bjc.6605904, PMID: 20842118 PMC2967071

[48] Hu F Li G Huang C Hou Z Yang X Luo X. The autophagy-independent role of BECN1 in colorectal cancer metastasis through regulating STAT3 signaling pathway activation. Cell Death Dis. (2020) 11:304. doi: 10.1038/s41419-020-2467-3, PMID: 32358527 PMC7195408

[49] Chen D Li Y Zhang X Wu H Wang Q Cai J. Ubiquitin ligase TRIM65 promotes colorectal cancer metastasis by targeting ARHGAP35 for protein degradation. Oncogene. (2019) 38:6429–44. doi: 10.1038/s41388-019-0891-6, PMID: 31332286 PMC6756236

[50] Lu Q Zhang Y Ma L Li D Li M Liu P. TRIM3 negatively regulates autophagy through promoting degradation of beclin1 in ewing sarcoma cells. Onco Targets Ther. (2019) 12:11587–95. doi: 10.2147/OTT.S219777, PMID: 32021240 PMC6942252

[51] Muesch A Hartmann E Rohde K Rubartelli A Sitia R Rapoport TA. A novel pathway for secretory proteins? Trends Biochem Sci. (1990) 15:86–8. doi: 10.1016/0968-0004(90)90186-f, PMID: 2139259

[52] Janvier K Bonifacino JS. Role of the endocytic machinery in the sorting of lysosome-associated membrane proteins. Mol Biol Cell. (2005) 16:4231–42. doi: 10.1091/mbc.e05-03-0213, PMID: 15987739 PMC1196333

[53] Ecard J Lian YL Divoux S Gouveia Z Vigne E Perez F. Lysosomal membrane proteins LAMP1 and LIMP2 are segregated in the Golgi apparatus independently of their clathrin adaptor binding motif. Mol Biol Cell. (2024) 35:ar42. doi: 10.1091/mbc.E23-06-0251, PMID: 38231876 PMC10916873

[54] O’Brien CE Bonanno L Zhang H Wyss-Coray T. Beclin 1 regulates neuronal transforming growth factor-β signaling by mediating recycling of the type I receptor ALK5. Mol Neurodegener. (2015) 10:69. doi: 10.1186/s13024-015-0065-0, PMID: 26692002 PMC4687091

[55] Ye SB Li ZL Luo DH Huang BJ Chen YS Zhang XS. Tumor-derived exosomes promote tumor progression and T-cell dysfunction through the regulation of enriched exosomal microRNAs in human nasopharyngeal carcinoma. Oncotarget. (2014) 5:5439–52. doi: 10.18632/oncotarget.2118, PMID: 24978137 PMC4170615

[56] Sun J Jia H Bao X Wu Y Zhu T Li R. Tumor exosome promotes Th17 cell differentiation by transmitting the lncRNA CRNDE-h in colorectal cancer. Cell Death Dis. (2021) 12:123. doi: 10.1038/s41419-020-03376-y, PMID: 33495437 PMC7835218

[57] Lloyd-Lewis B Krueger CC Sargeant TJ D’Angelo ME Deery MJ Feret R. Stat3-mediated alterations in lysosomal membrane protein composition. J Biol Chem. (2018) 293:4244–61. doi: 10.1074/jbc.RA118.001777, PMID: 29343516 PMC5868265

[58] Han Q Deng Y Chen S Chen R Yang M Zhang Z. Downregulation of ATG5-dependent macroautophagy by chaperone-mediated autophagy promotes breast cancer cell metastasis. Sci Rep. (2017) 7:4759. doi: 10.1038/s41598-017-04994-x, PMID: 28684853 PMC5500507

[59] Yu M Gou Wf Zhao S Xiao Lj Mao Xy Xing Yn. Beclin 1 expression is an independent prognostic factor for gastric carcinomas. Tumor Biol. (2013) 34:1071–83. doi: 10.1007/s13277-013-0648-8, PMID: 23334926

[60] Li BX Li CY Peng RQ Wu XJ Wang HY Wan DS. The expression of beclin 1 is associated with favorable prognosis in stage IIIB colon cancers. Autophagy. (2009) 5:303–6. doi: 10.4161/auto.5.3.7491, PMID: 19066461

[61] Zhang MY Wang LY Zhao S Guo XC Xu YQ Zheng ZH. Effects of beclin 1 overexpression on aggressive phenotypes of colon cancer cells. Oncol Lett. (2019) 17:2441–50. doi: 10.3892/ol.2018.9817, PMID: 30675309 PMC6341844

[62] Buratta S Tancini B Sagini K Delo F Chiaradia E Urbanelli L. Lysosomal exocytosis, exosome release and secretory autophagy: The autophagic- and endo-lysosomal systems go extracellular. Int J Mol Sci. (2020) 21:2576. doi: 10.3390/ijms21072576, PMID: 32276321 PMC7178086

[63] MaChado E White-Gilbertson S Van De Vlekkert D Janke L Moshiach S Campos Y. Regulated lysosomal exocytosis mediates cancer progression. Sci Adv. (2015) 1:e1500603. doi: 10.1126/sciadv.1500603, PMID: 26824057 PMC4730843

[64] MaChado ER Annunziata I van de Vlekkert D Grosveld GC d’Azzo A. Lysosomes and cancer progression: A Malignant liaison. Front Cell Dev Biol. (2021) 9. doi: 10.3389/fcell.2021.642494, PMID: 33718382 PMC7952443

[65] Lugli A Zlobec I Berger MD Kirsch R Nagtegaal ID. Tumour budding in solid cancers. Nat Rev Clin Oncol. (2021) 18:101–15. doi: 10.1038/s41571-020-0422-y, PMID: 32901132

[66] Bosch S Teerenstra S Wilt J Cunningham C Nagtegaal I. Predicting lymph node metastasis in PT1 colorectal cancer: a systematic review of risk factors providing rationale for therapy decisions. Endoscopy. (2013) 45:827–34. doi: 10.1055/s-0033-1344238, PMID: 23884793

[67] Ueno H Ishiguro M Nakatani E Ishikawa T Uetake H Matsuda C. Prospective multicenter study on the prognostic and predictive impact of tumor budding in stage II colon cancer: results from the SACURA trial. J Clin Oncol. (2019) 37:1886–94. doi: 10.1200/JCO.18.02059, PMID: 31180819 PMC6675595

[68] Bednarczyk M Muc-Wierzgoń M Dzięgielewska-Gęsiak S Fatyga E Waniczek D. Transcription of autophagy associated gene expression as possible predictors of a colorectal cancer prognosis. Biomedicines. (2023) 11:418. doi: 10.3390/biomedicines11020418, PMID: 36830954 PMC9952998

[69] Hu F Song D Yan Y Huang C Shen C Lan J. IL-6 regulates autophagy and chemotherapy resistance by promoting BECN1 phosphorylation. Nat Commun. (2021) 12:3651. doi: 10.1038/s41467-021-23923-1, PMID: 34131122 PMC8206314

[70] Denton D Nicolson S Kumar S. Cell death by autophagy: facts and apparent artefacts. Cell Death Differ. (2012) 19:87–95. doi: 10.1038/cdd.2011.146, PMID: 22052193 PMC3252836

[71] Guo B Tam A Santi SA Parissenti AM. Role of autophagy and lysosomal drug sequestration in acquired resistance to doxorubicin in MCF-7 cells. BMC Cancer. (2016) 16:762. doi: 10.1186/s12885-016-2790-3, PMID: 27687594 PMC5043608

[72] Fehrenbacher N Bastholm L Kirkegaard-Sørensen T Rafn B Bøttzauw T Nielsen C. Sensitization to the lysosomal cell death pathway by oncogene-induced down-regulation of lysosome-associated membrane proteins 1 and 2. Cancer Res. (2008) 68:6623–33. doi: 10.1158/0008-5472.CAN-08-0463, PMID: 18701486

[73] O’Donovan TR Rajendran S O’Reilly S O’Sullivan GC McKenna SL. Lithium modulates autophagy in esophageal and colorectal cancer cells and enhances the efficacy of therapeutic agents *in vitro* and *in vivo* . PloS One. (2015) 10:e0134676. doi: 10.1371/journal.pone.0134676, PMID: 26248051 PMC4527721

