# Supplementary material for: Implication of LAMP proteins and autophagy markers in colorectal cancer aggressiveness
Source: Front Immunol. 2025 Sep 30;16:1662830. doi: 10.3389/fimmu.2025.1662830 (PMC12518110; doi:10.3389/fimmu.2025.1662830)
Supplement: Supplementary file 1 [file DataSheet1.docx]

**Supplementary Table S1.**

**(a) Uncorrected and Benjamini-Hochberg corrected p-values associated with Fisher-Freeman-Halton test of LAMP and autophagy markers expression in CRC and normal colon tissues.**

| **LAMP1**  **↓ labels (optional) ↓** | **↓ Uncorrected P-values ↓** | **Benjamini-Hochberg significance** | **Benjamini-Hochberg corrected P-value** |
| --- | --- | --- | --- |
| LAMP1_TF by LAMP1_TNC | 2,0175E-09 | **significant** | 3,02625E-08 |
| LAMP1_TP by LAMP1_TNC | 0,000001873 | **significant** | 1,40475E-05 |
| LAMP1_TS by LAMP1_TNC | 2,88261E-06 | **significant** | 1,44131E-05 |
| LAMP1_TP by LAMP1_TF | 1,48149E-05 | **significant** | 5,55559E-05 |
| LAMP1_TF by LAMP1_NC | 0,0000875 | **significant** | 0,0002625 |
| LAMP1_TF by LAMP1_NC CRC | 0,00030129 | **significant** | 0,000753225 |
| LAMP1_TS by LAMP1_NC | 0,00046981 | **significant** | 0,001006736 |
| LAMP1_TS by LAMP1_TF | 0,000704252 | **significant** | 0,001320472 |
| LAMP1_TS by LAMP1_ NC CRC | 0,00786428 | **significant** | 0,013107133 |
| LAMP1_TP by LAMP1_TS | 0,0649 | **not significant** | 0,09735 |
| LAMP1_TNC by LAMP1_NC | 0,07723 | **not significant** | 0,105313636 |
| LAMP1_TP by LAMP1_NC | 0,11973629 | **not significant** | 0,144180888 |
| LAMP1_TP by LAMP1_ NC CRC | 0,12495677 | **not significant** | 0,144180888 |
| LAMP1_TNC by LAMP1_ NC CRC | 0,463798 | **not significant** | 0,496926429 |
| LAMP1_ NC CRC by LAMP1_NC | 0,99999999 | **not significant** | 0,99999999 |

Abbreviations: tissue regions (TP- tumor parenchyma; TS- tumor stroma; TF- tumor front; NC CRC- normal colon distal to the CRC area; NC- normal colon from nontumorous patients).

| **LAMP2**  **↓ labels (optional) ↓** | **↓ Uncorrected P-values ↓** | **Benjamini-Hochberg significance** | **Benjamini-Hochberg corrected P-value** |
| --- | --- | --- | --- |
| LAMP2_TF by LAMP2_TNC | 1,21174E-18 | **significant** | 1,81761E-17 |
| LAMP2_TS by LAMP2_TNC | 1,18689E-13 | **significant** | 8,90164E-13 |
| LAMP2_TP by LAMP2_TF | 2,50191E-09 | **significant** | 1,04508E-08 |
| LAMP2_TP by LAMP2_TNC | 2,78689E-09 | **significant** | 1,04508E-08 |
| LAMP2_TF by LAMP2_NC | 4,37715E-06 | **significant** | 1,31315E-05 |
| LAMP2_TS by LAMP2_NC | 8,17068E-05 | **significant** | 0,000204267 |
| LAMP2_TS by LAMP2_TF | 0,000306453 | **significant** | 0,000656685 |
| LAMP2_TNC by LAMP2_ NC CRC | 0,000783694 | **significant** | 0,001469426 |
| LAMP2_TF by LAMP2_ NC CRC | 0,000918832 | **significant** | 0,001531386 |
| LAMP2_TP by LAMP2_ NC CRC | 0,003025244 | **significant** | 0,004537866 |
| LAMP2_TS by LAMP2_ NC CRC | 0,014714023 | **significant** | 0,018415046 |
| LAMP2_TP by LAMP2_NC | 0,014732037 | **significant** | 0,018415046 |
| LAMP2_TP by LAMP2_TS | 0,02363208 | **significant** | 0,027267785 |
| LAMP2_ NC CRC by LAMP2_NC | 0,03216374 | **significant** | 0,03446115 |
| LAMP2_TNC by LAMP2_NC | 0,393556393 | **not significant** | 0,393556393 |

Abbreviations: tissue regions (TP- tumor parenchyma; TS- tumor stroma; TF- tumor front; NC CRC- normal colon distal to the CRC area; NC- normal colon from nontumorous patients.

| **LAMP2A**  **↓ labels (optional) ↓** | **↓ Uncorrected P-values ↓** | **Benjamini-Hochberg significance** | **Benjamini-Hochberg corrected P-value** |
| --- | --- | --- | --- |
| LAMP2A_TF by LAMP2A_TNC | 8,60481E-12 | **significant** | 1,29072E-10 |
| LAMP2A_TP by LAMP2A_TF | 1,15731E-08 | **significant** | 8,67986E-08 |
| LAMP2A_TP by LAMP2A_TNC | 1,22464E-07 | **significant** | 4,98159E-07 |
| LAMP2A_TS by LAMP2A_TF | 1,32843E-07 | **significant** | 4,98159E-07 |
| LAMP2A_TNC by LAMP2A_ NC CRC | 0,00017058 | **significant** | 0,000459601 |
| LAMP2A_TNC by LAMP2A_NC | 0,00018384 | **significant** | 0,000459601 |
| LAMP2A_TP by LAMP2A_TS | 0,000666841 | **significant** | 0,001428944 |
| LAMP2A_TP by LAMP2A_ NC CRC | 0,011214504 | **significant** | 0,019618157 |
| LAMP2A_TS by LAMP2A_NC | 0,011770894 | **significant** | 0,019618157 |
| LAMP2A_TF by LAMP2A_NC | 0,016012355 | **significant** | 0,023858053 |
| LAMP2A_TS by LAMP2A_ NC CRC | 0,017495906 | **significant** | 0,023858053 |
| LAMP2A_ NC CRC by LAMP2A_NC | 0,116883116 | **not significant** | 0,146103895 |
| LAMP2A_TS by LAMP2A_TNC | 0,22708433 | **not significant** | 0,262020381 |
| LAMP2A_TF by LAMP2A_ NC CRC | 0,473155698 | **not significant** | 0,506952534 |
| LAMP2A_TP by LAMP2A_NC | 0,731330348 | **not significant** | 0,731330348 |

Abbreviations: tissue regions (TP- tumor parenchyma; TS- tumor stroma; TF- tumor front; NC CRC- normal colon distal to the CRC area; NC- normal colon from nontumorous patients).

| **BECLIN1**  **↓ labels (optional) ↓** | **↓ Uncorrected P-values ↓** | **Benjamini-Hochberg significance** | **Benjamini-Hochberg corrected P-value** |
| --- | --- | --- | --- |
| BECLIN1_TP by BECLIN1_TS | 1,65537E-14 | **significant** | 2,48306E-13 |
| BECLIN1_TS by BECLIN1_TF | 1,19866E-11 | **significant** | 8,98992E-11 |
| BECLIN1_TS by BECLIN1_TNC | 7,10804E-08 | **significant** | 3,55402E-07 |
| BECLIN1_TP by BECLIN1_TF | 1,91172E-07 | **significant** | 7,16893E-07 |
| BECLIN1_TP by BECLIN1_TNC | 7,31357E-06 | **significant** | 2,19407E-05 |
| BECLIN1_TS by BECLIN1_ NC CRC | 0,000465301 | **significant** | 0,001163252 |
| BECLIN1_TS by BECLIN1_NC | 0,000938899 | **significant** | 0,002011927 |
| BECLIN1_TP by BECLIN1_NC | 0,015854778 | **significant** | 0,029727708 |
| BECLIN1_TP by BECLIN1_ NC CRC | 0,08162799 | **not significant** | 0,13604665 |
| BECLIN1_TF by BECLIN1_ NC CRC | 0,105040903 | **not significant** | 0,157561355 |
| BECLIN1_ NC CRC by BECLIN1_NC | 0,2007992 | **not significant** | 0,259553545 |
| BECLIN1_TF by BECLIN1_TNC | 0,207642836 | **not significant** | 0,259553545 |
| BECLIN1_TNC by BECLIN1_NC | 0,521234299 | **not significant** | 0,601424191 |
| BECLIN1_TNC by BECLIN1_ NC CRC | 0,573978158 | **not significant** | 0,614976598 |
| BECLIN1_TF by BECLIN1_NC | 1 | **not significant** | 1 |

Abbreviations: tissue regions (TP- tumor parenchyma; TS- tumor stroma; TF- tumor front; TNC- normal colon tissue in the CRC area; NC CRC- normal colon distal to the CRC area; NC- normal colon from nontumorous patients).

| **LC3B**  **↓ labels (optional) ↓** | **↓ Uncorrected P-values ↓** | **Benjamini-Hochberg significance** | **Benjamini-Hochberg corrected P-value** |
| --- | --- | --- | --- |
| LC3B_TF by LC3B_TNC | 5,41254E-05 | **significant** | 0,000541254 |
| LC3B_TP by LC3B_TNC | 0,000119076 | **significant** | 0,00059538 |
| LC3B_TNC by LC3B_ NC CRC | 0,000552079 | **significant** | 0,001840265 |
| LC3B_TS by LC3B_TNC | 0,001093334 | **significant** | 0,002733335 |
| LC3B_TP by LC3B_ NC CRC | 0,007859014 | **significant** | 0,015718028 |
| LC3B_TF by LC3B_ NC CRC | 0,01127974 | **significant** | 0,018799567 |
| LC3B_TS by LC3B_ NC CRC | 0,918162333 | **not significant** | 0,918162333 |
| LC3B_TP by LC3B_TF | 0,057275542 | **not significant** | 0,076425123 |
| LC3B_TS by LC3B_TF | 0,061140098 | **not significant** | 0,076425123 |
| LC3B_TP by LC3B_TS | 0,102405335 | **not significant** | 0,113783705 |
| LC3B_TS by LC3B_NC CRC | 0,918162333 | **not significant** | 0,918162333 |

Abbreviations: tissue regions (TP- tumor parenchyma; TS- tumor stroma; TF- tumor front; TNC- normal colon tissue in the CRC area; NC CRC- normal colon distal to the CRC area; NC- normal colon from nontumorous patients).

**(b) Uncorrected and Benjamini-Hochberg corrected p-values associated with Kendall`s tau correlation matrix of LAMP and autophagy markers expression with other clinicopathological characteristics in CRC and normal colon tissues.**

| **↓ labels (optional) ↓** | **↓ Uncorrected P-values ↓** | **Benjamini-Hochberg significance** | **Benjamini-Hochberg corrected P-value** |
| --- | --- | --- | --- |
| **LAMP1_TP - LAMP1_TF** | **0.000003** | **significant** | **0.000324** |
| **LAMP2A_TP - LAMP2A_TF** | **0.000003** | **significant** | **0.000324** |
| **LAMP2A_TS - LAMP2A_TF** | **0.000007** | **significant** | **0.000504** |
| **LAMP2_TP - LAMP2_TS** | **0.000045** | **significant** | **0.00243** |
| **LAMP2A_TP - LAMP2A_TS** | **0.000144** | **significant** | **0.004937143** |
| **LAMP2_TP - LAMP2_TF** | **0.000154** | **significant** | **0.004937143** |
| **pN - Budd** | **0.00016** | **significant** | **0.004937143** |
| **LAMP1_TS - LAMP1_TF** | **0.000326** | **significant** | **0.008802** |
| **LAMP1_TP - LAMP1_TS** | **0.000396** | **significant** | **0.009504** |
| **BECLIN1_TP - BECLIN1_TF** | **0.000674** | **significant** | **0.0145584** |
| **LAMP2_TS - LAMP2_TF** | **0.000903** | **significant** | **0.017731636** |
| **LAMP1_TS - LAMP2_TS** | **0.001653** | **significant** | **0.029754** |
| **LAMP1_TS - LAMP2A_TS** | **0.002099** | **significant** | **0.034875692** |
| **V - Budd** | **0.002433** | **significant** | **0.037537714** |
| **pN - V** | **0.003106** | **significant** | **0.0447264** |
| LAMP1_TP - LAMP2_TS | 0.005678 | **not significant** | 0.076653 |
| LAMP1_TS - BECLIN1_TF | 0.016871 | **not significant** | 0.214360941 |
| G - pT | 0.018612 | **not significant** | 0.223344 |
| LAMP1_TP - LAMP2_TP | 0.022166 | **not significant** | 0.245646 |
| pT - pN | 0.022745 | **not significant** | 0.245646 |
| pN - LC3B_TS | 0.034136 | **not significant** | 0.351113143 |
| pT - LAMP1_TP | 0.040156 | **not significant** | 0.378967304 |
| pT - V | 0.040353 | **not significant** | 0.378967304 |
| G - Mutacii | 0.044143 | **not significant** | 0.397287 |
| pT - LAMP1_TF | 0.049232 | **not significant** | 0.425088 |
| Budd - LAMP2_TF | 0.05159 | **not significant** | 0.425088 |
| LAMP1_TF - LAMP2_TS | 0.053136 | **not significant** | 0.425088 |
| Mutacii - BECLIN1_TP | 0.059313 | **not significant** | 0.457557429 |
| MSS_MSI - LAMP1_TF | 0.061911 | **not significant** | 0.461130207 |
| LAMP2_TP - LAMP2A_TF | 0.06614 | **not significant** | 0.476208 |
| Mutacii - LAMP2A_TP | 0.070369 | **not significant** | 0.490313032 |
| LAMP1_TS - LAMP2A_TP | 0.073097 | **not significant** | 0.490962977 |
| MSS_MSI - LAMP2_TS | 0.076867 | **not significant** | 0.490962977 |
| LAMP2_TP - LAMP2A_TP | 0.083429 | **not significant** | 0.490962977 |
| LAMP1_TP - LAMP2_TF | 0.083661 | **not significant** | 0.490962977 |
| LAMP2A_TS - BECLIN1_TF | 0.084297 | **not significant** | 0.490962977 |
| pN - LC3B_TP | 0.085192 | **not significant** | 0.490962977 |
| V - Mutacii | 0.090175 | **not significant** | 0.490962977 |
| LAMP1_TS - BECLIN1_TP | 0.092155 | **not significant** | 0.490962977 |
| LAMP2_TP - LAMP2A_TS | 0.092768 | **not significant** | 0.490962977 |
| Mutacii - LAMP2A_TF | 0.095226 | **not significant** | 0.490962977 |
| V - LAMP1_TF | 0.097707 | **not significant** | 0.490962977 |
| Mutacii - BECLIN1_TF | 0.097738 | **not significant** | 0.490962977 |
| LC3B_TP - LC3B_TS | 0.100348 | **not significant** | 0.492617455 |
| LAMP1_TP - BECLIN1_TP | 0.114517 | **not significant** | 0.5496816 |
| G - LAMP2_TP | 0.117974 | **not significant** | 0.55396487 |
| BECLIN1_TF - LC3B_TP | 0.12663 | **not significant** | 0.575936471 |
| BECLIN1_TS - LC3B_TS | 0.132006 | **not significant** | 0.575936471 |
| LAMP2A_TP - LC3B_TS | 0.134817 | **not significant** | 0.575936471 |
| LAMP2A_TP - BECLIN1_TP | 0.134874 | **not significant** | 0.575936471 |
| Mutacii - LAMP2A_TS | 0.135985 | **not significant** | 0.575936471 |
| LAMP2_TP - LC3B_TS | 0.144127 | **not significant** | 0.587385509 |
| LAMP2_TF - LC3B_TS | 0.144127 | **not significant** | 0.587385509 |
| LAMP1_TF - LAMP2A_TF | 0.156471 | **not significant** | 0.624224291 |
| BECLIN1_TP - BECLIN1_TS | 0.158946 | **not significant** | 0.624224291 |
| LAMP2A_TP - LC3B_TF | 0.170904 | **not significant** | 0.635234034 |
| pT - LAMP2_TP | 0.171154 | **not significant** | 0.635234034 |
| Budd - LC3B_TS | 0.171246 | **not significant** | 0.635234034 |
| LAMP2_TS - BECLIN1_TS | 0.173513 | **not significant** | 0.635234034 |
| LAMP1_TS - LAMP2_TP | 0.185672 | **not significant** | 0.645241622 |
| pT - Budd | 0.18717 | **not significant** | 0.645241622 |
| LAMP2_TS - LAMP2A_TP | 0.18817 | **not significant** | 0.645241622 |
| Budd - BECLIN1_TF | 0.199306 | **not significant** | 0.645241622 |
| G - V | 0.200195 | **not significant** | 0.645241622 |
| MSS_MSI - LAMP1_TS | 0.210916 | **not significant** | 0.645241622 |
| pT - LAMP2_TF | 0.211559 | **not significant** | 0.645241622 |
| LAMP2_TS - BECLIN1_TP | 0.212506 | **not significant** | 0.645241622 |
| LAMP1_TF - LAMP2_TF | 0.216171 | **not significant** | 0.645241622 |
| LAMP1_TP - BECLIN1_TF | 0.21643 | **not significant** | 0.645241622 |
| G - BECLIN1_TF | 0.219236 | **not significant** | 0.645241622 |
| LAMP2_TP - LC3B_TF | 0.220671 | **not significant** | 0.645241622 |
| LAMP2_TF - LC3B_TF | 0.220671 | **not significant** | 0.645241622 |
| BECLIN1_TP - LC3B_TP | 0.220671 | **not significant** | 0.645241622 |
| pN - LAMP2_TF | 0.221055 | **not significant** | 0.645241622 |
| LAMP2_TF - BECLIN1_TP | 0.234887 | **not significant** | 0.668851013 |
| G - BECLIN1_TP | 0.237304 | **not significant** | 0.668851013 |
| LAMP2_TS - LAMP2A_TF | 0.238433 | **not significant** | 0.668851013 |
| LAMP2A_TS - BECLIN1_TS | 0.250784 | **not significant** | 0.694478769 |
| LAMP2_TF - LAMP2A_TF | 0.254111 | **not significant** | 0.694784506 |
| LAMP1_TF - BECLIN1_TF | 0.258476 | **not significant** | 0.69749561 |
| V - LAMP2_TP | 0.264181 | **not significant** | 0.69749561 |
| G - LAMP2_TF | 0.26479 | **not significant** | 0.69749561 |
| LAMP2A_TS - LC3B_TS | 0.275947 | **not significant** | 0.714546 |
| LAMP1_TF - LC3B_TS | 0.277879 | **not significant** | 0.714546 |
| BECLIN1_TF - LC3B_TF | 0.285049 | **not significant** | 0.724359812 |
| LAMP1_TP - LC3B_TP | 0.288844 | **not significant** | 0.725468651 |
| LAMP1_TP - LAMP2A_TP | 0.296491 | **not significant** | 0.736115586 |
| LAMP2_TS - LAMP2A_TS | 0.306521 | **not significant** | 0.745174174 |
| pN - LAMP1_TS | 0.312829 | **not significant** | 0.745174174 |
| BECLIN1_TP - LC3B_TS | 0.314305 | **not significant** | 0.745174174 |
| G - LAMP2A_TS | 0.315039 | **not significant** | 0.745174174 |
| V - LAMP1_TP | 0.317389 | **not significant** | 0.745174174 |
| Mutacii - LAMP2_TF | 0.331202 | **not significant** | 0.763785095 |
| LAMP2_TF - LAMP2A_TP | 0.335664 | **not significant** | 0.763785095 |
| Budd - LC3B_TP | 0.335924 | **not significant** | 0.763785095 |
| G - LAMP1_TF | 0.344939 | **not significant** | 0.766267485 |
| LAMP1_TF - LC3B_TF | 0.347654 | **not significant** | 0.766267485 |
| LAMP1_TS - LAMP2_TF | 0.355108 | **not significant** | 0.766267485 |
| V - LAMP2_TF | 0.356664 | **not significant** | 0.766267485 |
| Budd - LAMP2A_TP | 0.358215 | **not significant** | 0.766267485 |
| LAMP2A_TP - BECLIN1_TF | 0.358301 | **not significant** | 0.766267485 |
| Budd - LAMP1_TF | 0.364615 | **not significant** | 0.770731615 |
| LAMP1_TS - LC3B_TP | 0.371093 | **not significant** | 0.770731615 |
| LC3B_TS - LC3B_TF | 0.371093 | **not significant** | 0.770731615 |
| LAMP1_TS - LAMP2A_TF | 0.379411 | **not significant** | 0.780502629 |
| pT - BECLIN1_TS | 0.404003 | **not significant** | 0.798406726 |
| pT - Mutacii | 0.407642 | **not significant** | 0.798406726 |
| LAMP2_TS - LC3B_TS | 0.410027 | **not significant** | 0.798406726 |
| Budd - BECLIN1_TP | 0.410685 | **not significant** | 0.798406726 |
| Mutacii - LAMP1_TP | 0.412053 | **not significant** | 0.798406726 |
| LC3B_TP - LC3B_TF | 0.414216 | **not significant** | 0.798406726 |
| V - LAMP2A_TP | 0.417554 | **not significant** | 0.798406726 |
| LAMP2A_TS - BECLIN1_TP | 0.417685 | **not significant** | 0.798406726 |
| pT - LAMP2_TS | 0.423086 | **not significant** | 0.801636632 |
| LAMP1_TP - BECLIN1_TS | 0.450369 | **not significant** | 0.843992069 |
| BECLIN1_TP - LC3B_TF | 0.453255 | **not significant** | 0.843992069 |
| LAMP2A_TS - LC3B_TF | 0.464214 | **not significant** | 0.852653647 |
| MSS_MSI - LAMP2_TF | 0.468816 | **not significant** | 0.852653647 |
| G - LAMP2_TS | 0.469749 | **not significant** | 0.852653647 |
| G - Budd | 0.475128 | **not significant** | 0.853626732 |
| V - LAMP2A_TS | 0.47855 | **not significant** | 0.853626732 |
| pN - LAMP2A_TF | 0.484168 | **not significant** | 0.853626732 |
| MSS_MSI - LAMP2_TP | 0.486093 | **not significant** | 0.853626732 |
| pN - LAMP2A_TS | 0.502869 | **not significant** | 0.868988736 |
| pN - MSS_MSI | 0.502887 | **not significant** | 0.868988736 |
| LAMP2A_TP - BECLIN1_TS | 0.521564 | **not significant** | 0.881375362 |
| LAMP1_TF - LC3B_TP | 0.524518 | **not significant** | 0.881375362 |
| pN - LAMP2_TP | 0.530556 | **not significant** | 0.881375362 |
| V - BECLIN1_TS | 0.535873 | **not significant** | 0.881375362 |
| pT - BECLIN1_TF | 0.545114 | **not significant** | 0.881375362 |
| LAMP1_TP - LC3B_TS | 0.546621 | **not significant** | 0.881375362 |
| LAMP2A_TF - BECLIN1_TF | 0.547771 | **not significant** | 0.881375362 |
| BECLIN1_TF - LC3B_TS | 0.550097 | **not significant** | 0.881375362 |
| Budd - LAMP1_TS | 0.563776 | **not significant** | 0.881375362 |
| MSS_MSI - BECLIN1_TS | 0.564179 | **not significant** | 0.881375362 |
| G - BECLIN1_TS | 0.565268 | **not significant** | 0.881375362 |
| LAMP1_TP - LAMP2A_TS | 0.570877 | **not significant** | 0.881375362 |
| Budd - LAMP2A_TS | 0.574124 | **not significant** | 0.881375362 |
| Budd - LAMP2_TS | 0.581138 | **not significant** | 0.881375362 |
| LAMP2_TF - BECLIN1_TS | 0.58122 | **not significant** | 0.881375362 |
| LAMP2A_TF - LC3B_TS | 0.583882 | **not significant** | 0.881375362 |
| LAMP2A_TF - BECLIN1_TP | 0.587799 | **not significant** | 0.881375362 |
| LAMP1_TP - LC3B_TF | 0.591505 | **not significant** | 0.881375362 |
| pN - LC3B_TF | 0.598161 | **not significant** | 0.881375362 |
| V - LAMP2A_TF | 0.601036 | **not significant** | 0.881375362 |
| G - LAMP1_TP | 0.608839 | **not significant** | 0.881375362 |
| G - MSS_MSI | 0.615212 | **not significant** | 0.881375362 |
| LAMP2A_TF - LC3B_TP | 0.617075 | **not significant** | 0.881375362 |
| BECLIN1_TS - LC3B_TP | 0.617075 | **not significant** | 0.881375362 |
| LAMP1_TF - BECLIN1_TP | 0.620703 | **not significant** | 0.881375362 |
| G - pN | 0.626402 | **not significant** | 0.881375362 |
| pN - Mutacii | 0.627148 | **not significant** | 0.881375362 |
| MSS_MSI - LAMP2A_TF | 0.629691 | **not significant** | 0.881375362 |
| pT - LAMP2A_TF | 0.642123 | **not significant** | 0.881375362 |
| V - LAMP1_TS | 0.650506 | **not significant** | 0.881375362 |
| LAMP2_TP - BECLIN1_TF | 0.651095 | **not significant** | 0.881375362 |
| Budd - Mutacii | 0.659032 | **not significant** | 0.881375362 |
| LAMP1_TF - LAMP2_TP | 0.663281 | **not significant** | 0.881375362 |
| pT - LAMP2A_TS | 0.673224 | **not significant** | 0.881375362 |
| LAMP2_TP - BECLIN1_TS | 0.677561 | **not significant** | 0.881375362 |
| pT - LAMP2A_TP | 0.678597 | **not significant** | 0.881375362 |
| pN - LAMP2A_TP | 0.680739 | **not significant** | 0.881375362 |
| MSS_MSI - LAMP2A_TS | 0.680884 | **not significant** | 0.881375362 |
| LAMP2A_TS - LC3B_TP | 0.690328 | **not significant** | 0.881375362 |
| LAMP2_TP - BECLIN1_TP | 0.691527 | **not significant** | 0.881375362 |
| LAMP2_TS - LC3B_TF | 0.692633 | **not significant** | 0.881375362 |
| pN - LAMP1_TF | 0.693684 | **not significant** | 0.881375362 |
| pT - MSS_MSI | 0.695377 | **not significant** | 0.881375362 |
| LAMP2_TS - BECLIN1_TF | 0.696276 | **not significant** | 0.881375362 |
| BECLIN1_TS - BECLIN1_TF | 0.703791 | **not significant** | 0.881375362 |
| Budd - LAMP2_TP | 0.70578 | **not significant** | 0.881375362 |
| pN - LAMP1_TP | 0.706457 | **not significant** | 0.881375362 |
| LAMP1_TS - BECLIN1_TS | 0.707157 | **not significant** | 0.881375362 |
| Mutacii - LAMP1_TF | 0.715536 | **not significant** | 0.881375362 |
| LAMP2_TF - LAMP2A_TS | 0.717563 | **not significant** | 0.881375362 |
| pN - BECLIN1_TP | 0.736274 | **not significant** | 0.881375362 |
| MSS_MSI - LAMP1_TP | 0.737449 | **not significant** | 0.881375362 |
| LAMP2_TP - LC3B_TP | 0.738883 | **not significant** | 0.881375362 |
| LAMP2_TF - LC3B_TP | 0.738883 | **not significant** | 0.881375362 |
| MSS_MSI - LAMP2A_TP | 0.742855 | **not significant** | 0.881375362 |
| V - MSS_MSI | 0.74292 | **not significant** | 0.881375362 |
| V - BECLIN1_TP | 0.750288 | **not significant** | 0.881375362 |
| Mutacii - LAMP1_TS | 0.752116 | **not significant** | 0.881375362 |
| LAMP2A_TF - LC3B_TF | 0.759463 | **not significant** | 0.881375362 |
| BECLIN1_TS - LC3B_TF | 0.759463 | **not significant** | 0.881375362 |
| pN - BECLIN1_TS | 0.763173 | **not significant** | 0.881375362 |
| pN - BECLIN1_TF | 0.763859 | **not significant** | 0.881375362 |
| LAMP2_TF - BECLIN1_TF | 0.767123 | **not significant** | 0.881375362 |
| LAMP1_TS - LC3B_TF | 0.777297 | **not significant** | 0.884250568 |
| Budd - BECLIN1_TS | 0.777813 | **not significant** | 0.884250568 |
| LAMP1_TP - LAMP2A_TF | 0.794421 | **not significant** | 0.898402806 |
| Mutacii - BECLIN1_TS | 0.801917 | **not significant** | 0.902156625 |
| G - LAMP2A_TF | 0.807613 | **not significant** | 0.903857036 |
| Budd - LC3B_TF | 0.813664 | **not significant** | 0.905935175 |
| LAMP2_TS - LC3B_TP | 0.829638 | **not significant** | 0.918983631 |
| Budd - LAMP1_TP | 0.845861 | **not significant** | 0.932173347 |
| V - LAMP2_TS | 0.853489 | **not significant** | 0.935805198 |
| pN - LAMP2_TS | 0.871282 | **not significant** | 0.945162783 |
| MSS_MSI - BECLIN1_TF | 0.87654 | **not significant** | 0.945162783 |
| LAMP2A_TF - BECLIN1_TS | 0.880191 | **not significant** | 0.945162783 |
| V - BECLIN1_TF | 0.882146 | **not significant** | 0.945162783 |
| MSS_MSI - BECLIN1_TP | 0.893885 | **not significant** | 0.945162783 |
| LAMP1_TS - LC3B_TS | 0.898669 | **not significant** | 0.945162783 |
| Budd - LAMP2A_TF | 0.90017 | **not significant** | 0.945162783 |
| G - LAMP1_TS | 0.901722 | **not significant** | 0.945162783 |
| G - LAMP2A_TP | 0.903843 | **not significant** | 0.945162783 |
| Mutacii - LAMP2_TP | 0.905781 | **not significant** | 0.945162783 |
| Mutacii - MSS_MSI | 0.920595 | **not significant** | 0.9560025 |
| LAMP1_TF - LAMP2A_TS | 0.929825 | **not significant** | 0.960967464 |
| Mutacii - LAMP2_TS | 0.94248 | **not significant** | 0.965408417 |
| pT - LAMP1_TS | 0.943061 | **not significant** | 0.965408417 |
| Budd - MSS_MSI | 0.94892 | **not significant** | 0.966669972 |
| LAMP1_TF - BECLIN1_TS | 0.953244 | **not significant** | 0.966669972 |
| pT - BECLIN1_TP | 0.97647 | **not significant** | 0.985595888 |
| LAMP1_TF - LAMP2A_TP | 1 | **not significant** | 1 |
| LAMP2A_TP - LC3B_TP | 1 | **not significant** | 1 |

Abbreviations: tissue regions (pN- invasion in lymph nodes; V-blood vessels; TP- tumor parenchyma; TS- tumor stroma; TF- tumor front; NC CRC- normal colon distal to the CRC area; NC- normal colon from nontumorous patients).

**(c) Uncorrected and Benjamini-Hochberg corrected p-values associated with Kendall`s tau correlation matrix of LAMP and autophagy markers expression in CRC blood samples (plasma and WBC) and other clinical variables.**

| **↓ labels (optional) ↓** | **↓ Uncorrected P-values ↓** | **Benjamini-Hochberg significance** | **Benjamini-Hochberg corrected P-value** |
| --- | --- | --- | --- |
| BECLIN1_mRNA_WBC-LC3B_mRNA_WBC | **0.000005** | **significant** | **0.00018** |
| LAMP2_mRNA_WBC-LC3B_mRNA_WBC | **0.000013** | **significant** | **0.000204** |
| LAMP2_mRNA_WBC-BECLIN1_mRNA_WBC | **0.000017** | **significant** | **0.000204** |
| LAMP1_PLASMA- pN | 0.01302 | **not significant** | 0.11718 |
| LAMP1_PLASMA- BECLIN1_PLASMA | 0.030945 | **not significant** | 0.222804 |
| BECLIN1_PLASMA- pN | 0.0423 | **not significant** | 0.2538 |
| pT - BECLIN1_mRNA_WBC | 0.095058 | **not significant** | 0.312437455 |
| pT - LAMP1_mRNA_WBC | 0.095108 | **not significant** | 0.312437455 |
| pT - LAMP2_mRNA_WBC | 0.095108 | **not significant** | 0.312437455 |
| pT - LC3B_mRNA_WBC | 0.095108 | **not significant** | 0.312437455 |
| pT - LAMP1_PLASMA | 0.095467 | **not significant** | 0.312437455 |
| pT - LAMP2_PLASMA | 0.205275 | **not significant** | 0.615825 |
| pN - LAMP1_mRNA_WBC | 0.257757 | **not significant** | 0.670451143 |
| LAMP1_PLASMA - LAMP1_mRNA_WBC | 0.260731 | **not significant** | 0.670451143 |
| LAMP1_PLASMA - LAMP2_PLASMA | 0.299989 | **not significant** | 0.69811425 |
| LAMP1_PLASMA - BECLIN1_mRNA_WBC | 0.310273 | **not significant** | 0.69811425 |
| BECLIN1_mRNA_WBC - BECLIN1_PLASMA | 0.424138 | **not significant** | 0.8970534 |
| pT - pN | 0.476661 | **not significant** | 0.8970534 |
| LAMP1_mRNA_WBC - BECLIN1_mRNA_WBC | 0.478356 | **not significant** | 0.8970534 |
| pT - BECLIN1_PLASMA | 0.498363 | **not significant** | 0.8970534 |
| LAMP2_PLASMA - BECLIN1_mRNA_WBC | 0.566496 | **not significant** | 0.900408 |
| LAMP1_mRNA_WBC - BECLIN1_PLASMA | 0.568066 | **not significant** | 0.900408 |
| pN - BECLIN1_mRNA_WBC | 0.580402 | **not significant** | 0.900408 |
| LAMP2_PLASMA - LC3B_mRNA_WBC | 0.600272 | **not significant** | 0.900408 |
| LC3B_mRNA_WBC - BECLIN1_PLASMA | 0.731943 | **not significant** | 0.934090839 |
| pN - LAMP2_mRNA_WBC | 0.732242 | **not significant** | 0.934090839 |
| LAMP2_PLASMA - LAMP2_mRNA_WBC | 0.760002 | **not significant** | 0.934090839 |
| pN - LC3B_mRNA_WBC | 0.772193 | **not significant** | 0.934090839 |
| LAMP2_PLASMA - BECLIN1_PLASMA | 0.803697 | **not significant** | 0.934090839 |
| LAMP1_mRNA_WBC - LAMP2_mRNA_WBC | 0.804356 | **not significant** | 0.934090839 |
| LAMP1_mRNA_WBC - LC3B_mRNA_WBC | 0.804356 | **not significant** | 0.934090839 |
| LAMP1_PLASMA - LAMP2_mRNA_WBC | 0.947253 | **not significant** | 0.982406 |
| pN - LAMP2_PLASMA | 0.956011 | **not significant** | 0.982406 |
| LAMP2_PLASMA - LAMP1_mRNA_WBC | 0.965203 | **not significant** | 0.982406 |
| LAMP2_mRNA_WBC - BECLIN1_PLASMA | 0.96964 | **not significant** | 0.982406 |
| LAMP1_PLASMA - LC3B_mRNA_WBC | 0.982406 | **not significant** | 0.982406 |

Abbreviations: (pT- tumor stage; pN- invasion in lymph nodes).
